# A comparison of arm‐based and contrast‐based models for network meta‐analysis

**DOI:** 10.1002/sim.8360

**Published:** 2019-10-03

**Authors:** Ian R. White, Rebecca M. Turner, Amalia Karahalios, Georgia Salanti

**Affiliations:** ^1^ MRC Clinical Trials Unit at UCL Institute of Clinical Trials and Methodology London UK; ^2^ School of Public Health and Preventive Medicine Monash University Melbourne Australia; ^3^ Institute of Social and Preventive Medicine University of Bern Bern Switzerland

**Keywords:** Bayesian, missing data, mixed treatment comparisons, multiple treatments meta‐analysis, network meta‐analysis

## Abstract

Differences between arm‐based (AB) and contrast‐based (CB) models for network meta‐analysis (NMA) are controversial. We compare the CB model of Lu and Ades (2006), the AB model of Hong et al(2016), and two intermediate models, using hypothetical data and a selected real data set. Differences between models arise primarily from study intercepts being fixed effects in the Lu‐Ades model but random effects in the Hong model, and we identify four key difference. (1) If study intercepts are fixed effects then only within‐study information is used, but if they are random effects then between‐study information is also used and can cause important bias. (2) Models with random study intercepts are suitable for deriving a wider range of estimands, eg, the marginal risk difference, when underlying risk is derived from the NMA data; but underlying risk is usually best derived from external data, and then models with fixed intercepts are equally good. (3) The Hong model allows treatment effects to be related to study intercepts, but the Lu‐Ades model does not. (4) The Hong model is valid under a more relaxed missing data assumption, that arms (rather than contrasts) are missing at random, but this does not appear to reduce bias. We also describe an AB model with fixed study intercepts and a CB model with random study intercepts. We conclude that both AB and CB models are suitable for the analysis of NMA data, but using random study intercepts requires a strong rationale such as relating treatment effects to study intercepts.

## 1 INTRODUCTION

Network meta‐analysis (NMA) aims to synthesize a body of evidence describing comparisons between multiple treatments or interventions for the same condition. It thus combines direct comparisons (where treatments are compared within a study) with indirect comparisons (where treatments are compared with a common comparator in different studies). Network meta‐analysis is increasingly popular, with 456 NMAs with four or more treatments identified up until 2015.^1^

Recent methodological developments in NMA have given rise to a debate about models that focus on arms versus models that focus on contrasts.^2^, ^3^, ^4^, ^5^ In NMA, *arm* refers to a single treatment group in a single study, and *contrast* refers to a relative treatment effect between arms using a suitable metric. The terms *contrast‐based* (CB) and *arm‐based* (AB) have been used in different ways to describe NMAs. Salanti et al described a CB NMA as a model for the set of estimated contrasts, and an AB NMA as a model for the raw arm‐level data.^6^ In this view, CB and AB models describe the data expressed in different ways, and so we refer to these as *a CB likelihood* and *an AB likelihood*.

Hong et al used the terms CB and AB in a different way: they used an AB likelihood and applied modeling assumptions either to parameters representing contrasts (“CB models”) or to parameters representing arm means (“AB models”).^3^ Taking a missing data perspective, with treatments not included in a study regarded as missing data, they argued that the AB models make better use of the data and reduce bias compared with other methods. Zhang et al focused on the estimands in meta‐analysis in the binary data case, and argued that the CB model is limited to specific estimands (quantities of interest) while the AB model offers a wider range of estimands.^2^ Other authors have used the terms CB and AB in a similar way but suggesting different models.^7^, ^8^

Dias and Ades criticized the work of Hong et al on several grounds, most notably that their AB model compromised randomization and was thus prone to bias.^4^ In response, Hong et al argued that the AB model makes a more credible assumption about the missing data.^5^

The aim of this article is to understand the differences between CB models and AB models. We do this by defining clear terminology and notation, which we hope will be used in future papers; discussing what quantity is being estimated (the estimand); exploring the impact of compromising randomization in these models; and exploring the missing data assumptions underlying analyses using these models. For simplicity, we consider a single binary outcome using the odds ratio metric under a consistency assumption (that indirect and direct comparisons estimate the same parameter^6^). The models are compared for AB likelihoods, which give greater modeling flexibility.

In Section 2, we describe and compare the models considered, including modeling of heterogeneity variances and use of appropriately informative priors. In Section 3, we discuss the estimands which can be estimated by each model. In Section 4, we explore the consequences of compromising randomization, using hypothetical data. In Section 5, we discuss the assumptions made by the different models from a missing data perspective, again using hypothetical data. In Section 6, we analyze a real network selected to illustrate the differences between the models. We conclude with a discussion, extensions, and some key messages in Section 7.

## 2 CONTRAST‐BASED AND ARM‐BASED MODELS

### 2.1 Notation

We follow standard statistical practice in using Greek letters for unknown parameters. Superscripts *a* and *c* identify quantities that relate to arms and to contrasts between arms, respectively. *N*(*μ*,*σ* ^2^) denotes a Normal distribution with mean *μ* and standard deviation *σ*. The term “fixed effect” denotes a parameter which is to be estimated freely, unlike a “random effect,” which shares a distribution with other parameters. The “common‐effect” model denotes the meta‐analysis model with no heterogeneity.^9^ Notation involving *μ* and *σ* ^2^ or **Σ** (defined in detail below) denotes mean and variance across studies. Vectors and matrices are in bold font.

Let *i*=1,…,*n* denote study, and *k*=1,…,*K* denote treatment. Let *R*_*i*_ be the set of treatments in study *i*, which we call the study design.^10^ Let $\theta_{ik}^{a}$ be the parameter of interest in arm *k* of study *i*. The parameter $\theta_{ik}^{a}$ describes the data through the measurement model (Section 2.2) and is in turn described by the structural model (Section 2.3). In our binary outcome setting, the data are the number of participants *n*_*ik*_ and the number of successes $y_{ik}^{a}$ in arm *k* of study *i*, and $\theta_{ik}^{a}$ is the log odds of success; the natural effect measures are the (log) odds ratios, but it is possible to estimate other summaries (estimands) such as a risk difference at the population level (Section 3).

### 2.2 Measurement model: likelihood choices

Salanti et al described CB and AB NMA, which in our terminology are CB and AB likelihoods.^6^ The AB likelihood uses the arm‐level data $y_{ik}^{a}$ whose distribution is binomial with denominator the sample size *n*_*ik*_ and probability $h(\theta_{ik}^{a})$, where *h*(.) is the inverse logit function. An (exact) AB likelihood is based on this binomial distribution. Another possibility, little used in practice and not pursued here, is a Normal‐approximation AB likelihood based on a set of estimates ${\hat{\theta }}_{ik}^{a}$ and their variances.

The CB likelihood implies a two‐stage approach to estimation. In the first stage, the estimated log odds ratio $y_{ikk^{\prime }}^{c}$ comparing arms *k* and *k* *′* of study *i* is computed, together with its standard error *s*_*ikk′*_, from the arm‐level data. (Multiarm studies provide a vector of outcomes $\left(y_{ikk^{\prime }}^{c},y_{ikk^{\prime \prime }}^{c},\text{…}\right)$ with its variance‐covariance matrix). In the second stage, the estimated log odds ratios are analyzed using the Normal‐approximation likelihood $y_{ikk^{\prime }}^{c}∼N(\theta_{ik^{\prime }}^{a}-\theta_{ik}^{a},s_{ikk^{\prime }}^{2})$, where *s*_*ikk′*_ is assumed known. The approximations involved tend to be good in meta‐analyses of large studies, when one‐stage and two‐stage approaches give very similar answers,^11^ but the approximations can cause bias with smaller studies.^12^

### 2.3 Structural models

We describe a sequence of four models for an AB likelihood, leading in steps from the widely used model of Lu and Ades^13^ (model 1) to the AB model proposed by Hong et al^3^(model 4). The intermediate models help to shed light on the important differences between models 1 and 4. Two of the models have both CB and AB forms.

#### 2.3.1 Model 1: CB model describing observed arms

We start with the CB model of Lu and Ades,^13^ which requires a study‐specific reference treatment *b*_*i*_ to be defined in each study *i*. This is also the model fitted by Salanti et al: to avoid confusion, we stress that their “AB NMA” in our terminology is model 1 with an AB likelihood.^6^ The model is

(1) $$\theta_{ik}^{a}=\alpha_{ib_{i}}^{a}+\delta_{ib_{i}k}^{c}\;\text{for}\;k\in R_{i}.$$

We call $\alpha_{ib_{i}}^{a}$ the study intercept: it is the log odds in arm *b*_*i*_ of study *i* and is a fixed effect. The study‐specific treatment contrast $\delta_{ib_{i}k}^{c}$ compares treatment *k* with *b*_*i*_, for *k*∈*R*_*i*_. We set $\delta_{ib_{i}k}^{c}=0$ if *k*=*b*_*i*_, and otherwise, we model

(2) $$\delta_{ib_{i}k}^{c}∼N\left(\mu_{1k}^{c}-\mu_{1b_{i}}^{c},\sigma^{c2}\right),$$

which incorporates the consistency assumption.^6^ The overall mean treatment effects $\mu_{1k}^{c}$ for *k*>1 compare each treatment *k* with the reference treatment 1 and are the key model parameters; we set $\mu_{11}^{c}=0$. *σ* ^*c*2^ in Equation (2) is the *contrast heterogeneity variance*; note that “*c*” is a superscript, but “2” is a power. In this model, the contrast heterogeneity variance is the same for all treatment contrasts. The model can be extended to allow heterogeneity variances to vary between treatment contrasts, as discussed in Section 2.4.

#### 2.3.2 Model 2: CB model describing all possible arms

We now modify model 1 by describing $\theta_{ik}^{a}$ in all arms, not just the observed arms

(3) $$\;\theta_{ik}^{a}=\alpha_{i1}^{a}+\delta_{i1k}^{c}$$

(4) $$\delta_{i}^{c}=\left(\delta_{i12}^{c},\text{…},\delta_{i1K}^{c}\right)∼N(\mu^{c},\sum^{c}),$$

where $\delta_{i11}^{c}=0$ and $\mu^{c}=(\mu_{12}^{c},\text{…},\mu_{1K}^{c})$ is the vector of overall mean effects for treatments 2,…,*k* compared with the reference treatment 1. The structure of the contrast heterogeneity variance matrix **Σ** ^*c*^ is discussed in Section 2.4. The off‐diagonal elements of **Σ** ^*c*^ are needed to define the heterogeneity variances for contrasts not involving treatment 1; for example, the *k*−*k* *′* contrast heterogeneity variance $\sum_{kk}^{c}-2\sum_{kk^{\prime }}^{c}+\sum_{k^{\prime }k^{\prime }}^{c}$. The study intercepts $\alpha_{i1}^{a}$ now all refer to treatment 1, even if 1∉*R*_*i*_; if treatment 1 is a control treatment, then the study intercepts describe *underlying risk*.^14^ Model 2 embodies a useful alternative view of consistency, ie, that the treatment effects follow the same model in designs where they are unobserved as in designs where they are observed.

Modeling all arms, not just the observed arms, has no impact on the model fit. From a statistical point of view, therefore, there is no difference between models 1 and 2, provided they model heterogeneity variances in the same way: we prove this for Bayesian estimation in Supplementary Appendix A. In Bayesian computation using Monte Carlo Markov Chain methods, however, the extra unidentified parameters in model 2 may increase autocorrelation in the Markov Chain and hence decrease computational efficiency.

Closely related to model 2 is an AB model that handles the treatments symmetrically,^7^, ^15^

(5) $$\;\theta_{ik}^{a}=\alpha_{i}^{a}+\mu_{k}^{c}+\eta_{ik}^{a}$$

(6) $$\eta_{i}^{a}=\left(\eta_{i1}^{a},\text{…},\eta_{iK}^{a}\right)∼N(0,\sum^{a}).$$

In this model, we need one constraint on the fixed parameters, and we choose to set $\mu_{1}^{c}=0$ (which is why we write the treatment effects $\mu_{k}^{c}$ in model (5) as contrast parameters). We show in Supplementary Appendix A that model (5), (6) is equivalent to model (3), (4) under Bayesian estimation with flat priors for the study intercepts $\alpha_{i1}^{a}$, $\alpha_{i}^{a}$. This contrasts with frequentist estimation, where parameter estimates differ between models (3, 4) and (5, 6) and between different choices of reference treatments in model (3, 4).^15^ Improved frequentist estimation methods reduce these discrepancies.^8^

#### 2.3.3 Model 3: CB model with random study intercepts

Study intercepts were fixed effects in models 1 and 2. Model 3 modifies model 2 by making them random effects, so that, alongside Equations (3) and (4), we have

(7) $$\alpha_{i1}^{a}∼N\left(\mu_{1}^{a},\sigma^{a2}\right),$$

where we call *σ* ^*a*2^ the *arm heterogeneity variance* for the reference treatment; again, *a* is a superscript and 2 is a power.

The extra assumption in model 3 should lead to greater precision. However, this comes at the price of using “between‐study information,” meaning that the treatment effect estimated across the network is informed not only by the usual differences within studies but also by differences between studies; for example, if participants in studies containing treatment Y have worse outcomes (on all arms) than participants in studies containing an equally effective treatment Z, then treatment Y may appear worse than treatment Z.^16^ We explore this issue further in Section 4.

#### 2.3.4 Model 4: model with random study intercepts related to treatment effects

Model 3 assumes that the treatment effects $\delta_{i}^{c}$ in study *i* are independent of the study intercepts $\alpha_{i1}^{a}$. Model 4 relaxes this assumption. We first write model 4 in a CB form, where, alongside Equation (3) and replacing Equations (4) and (7), we have

(8) $$\left(\alpha_{i1}^{a},\delta_{i}^{c}\right)∼N(\mu^{*},\sum^{*}),$$

where $\mu^{*}={{\left(\mu_{1}^{a},(\mu^{c}\right)}^{T})}^{T}$ and **Σ** ^∗^ is a *K*×*K* variance matrix. Model 3 is the special case of model (8) with $\sum_{1k}^{*}=\sum_{k1}^{*}=0$ for all *k*>1. In the rest of this paper, we use the AB form of this model, which is the AB model of Hong et al^3^

(9) $$\theta_{i}^{a}={\left(\theta_{i1}^{a},\theta_{i2}^{a},\text{…},\theta_{iK}^{a}\right)}^{T}∼N(\mu^{a},\sum^{a}),$$

where the arm‐specific means $\mu^{a}={\left(\mu_{1}^{a},\mu_{2}^{a},\text{…},\mu_{K}^{a}\right)}^{T}$ are fixed effects and the parameters of interest are $\mu_{k}^{c}=\mu_{k}^{a}-\mu_{1}^{a}$ for *k*=2,…,*K*. The heterogeneity variance for the *k*−*k* *′* contrast is $\sum_{kk}^{a}-2\sum_{kk^{\prime }}^{a}+\sum_{k^{\prime }k^{\prime }}^{a}$. We discuss the structure of the heterogeneity variance **Σ** ^*a*^ in Section 2.4. In Supplementary Appendix B, we show that models (8) and (9) are equivalent.

### 2.4 Modeling heterogeneity variances

Model 1 using Equation (2) assumes the same heterogeneity variance *σ* ^*c*2^ for all treatment contrasts. We call this the common heterogeneity (CH) variance model. The remaining models naturally allow non‐CH (NCH) variances simply by imposing no constraints on **Σ** ^*c*^, **Σ** ^∗^, or **Σ** ^*a*^.

Model 1 can be extended to allow NCH,^17^ but we do not use this model since model 2 more conveniently allows NCH. In particular, the “second‐order consistency” assumptions proposed to improve precision of estimation in the NCH version of model 1^17^ are naturally implied by **Σ** ^*c*^ being positive semidefinite in model 2.

We can assume CH in models 2 and 3 by setting

(10) $$\sum^{c}=\sigma^{c2}P_{K-1}(0.5),$$

where ***P***_*n*_(*ρ*) is the *n*×*n* matrix with all diagonal elements equal to 1 and all off‐diagonal elements equal to *ρ*.^18^ Structured models for NCH are also possible.^13^, ^17^, ^19^ We can assume CH in the AB version of model 2 by setting

(11) $$\sum^{a}=0.5\sum^{c2}I_{K}$$

and structured models for NCH include diagonal and factor‐analytic models.^15^

Modeling the heterogeneity variance **Σ** ^*a*^ in model 4 requires care, since the matrix involves both arm heterogeneity and contrast heterogeneity. Common arm heterogeneity means that the $\sum_{kk}^{a}$ terms in model (9) are the same for all *k*, while common contrast heterogeneity means that the contrast variance $\sum_{kk}^{a}-2\sum_{kk^{\prime }}^{a}+\sum_{k^{\prime }k^{\prime }}^{a}$ is the same for all *k* and *k* *′* (1 ≤ *k*,*k* *′* ≤ *K*,*k* *′*≠*k*). For model 4 with CH, we therefore propose a compound symmetry structure, allowing separate parameters for the contrast heterogeneity and the arm heterogeneity:

(12) $$\sum^{a}=\sigma^{a2}P_{K}(\rho^{a}),$$

where *ρ* ^*a*^ is an unknown parameter. In this model, the variance for arm heterogeneity is *σ* ^*a*2^ and the variance for contrast heterogeneity is

(13) $$\sigma^{c2}=2\sigma^{a2}(1-\rho^{a}).$$

It is convenient to write the likelihood in terms of the correlation *ρ* ^*a*^ and the contrast heterogeneity variance *σ* ^*c*2^. In this model, the regression of treatment contrasts (treatment *k* versus 1) on underlying risk (treatment 1) has slope *ρ* ^*a*^−1, so *ρ* ^*a*^=1 indicates no association between treatment contrasts and underlying risk. Model 4 with CH therefore has the disadvantage that it cannot accommodate treatment contrasts being both heterogeneous and uncorrelated with underlying risk.

Hong et al^3^ proposed a diagonal form $\sum^{a}=diag(\sigma_{1}^{a2},\sigma_{2}^{a2},\text{…},\sigma_{K}^{a2})$; a special case has $\sigma_{k}^{a2}=\sigma^{a2}$ for all *k* and so $\sum^{a}=\sigma^{a2}I_{K}$, which is model (12) with *ρ* ^*a*^=0. These models imply that the contrast heterogeneities $\sigma_{k}^{a2}+\sigma_{k^{\prime }}^{a2}$ are greater than the arm heterogeneities $\sigma_{k}^{a2}$, whereas the opposite is likely to be true; hence, we do not pursue the diagonal form.

### 2.5 Choice of prior

We use Bayesian estimation of the aforementioned models because of its versatility and its ability to incorporate informative priors. We use evidence‐based priors for the contrast heterogeneity variance^20^; details, including details of how we make priors comparable across models, are given in Supplementary Appendix C. We use noninformative *N*(0,1000) priors for all other parameters. Other prior choices are of course possible. Stata code for fitting these models is given in Supplementary Appendix E.

## 3 ESTIMANDS

An estimand describes what is being estimated and in what population. In mixed‐effects logistic regression models, we distinguish marginal (population‐averaged) estimands from conditional (cluster‐specific) estimands^21^; conditional odds ratios tend to be further from 1 than marginal odds ratios. In NMA, the “cluster” is the study. The parameters $\mu_{k}^{c}$ in models 1 to 4 all represent the relative effect of an intervention on the odds within a single study (a conditional estimand). In later sections, we therefore compare the methods for estimating the conditional odds ratio. The marginal estimand, on the other hand, is the relative effect of an intervention across the whole population of studies, and need not be expressed as an odds ratio.^2^ The different types of summary have different uses. For example, if a NMA includes studies at different hospitals in a country, then a policy maker considering introducing a policy at a national level would be more interested in a marginal estimand, and specifically in the marginal risk difference, while a particular hospital would be more interested in a hospital‐specific (conditional) estimand.

Model 4 also allows estimation of marginal treatment means $\pi_{k}=E[\text{expit}(\theta_{ik}^{a})]$, where the expectation is across the heterogeneity distribution in Equation (9). Marginal contrasts $g(\pi_{k})-g(\pi_{1})$, where g(.) is a logistic or other link function, may then be obtained. This is straightforwardly implemented in Bayesian computation. A similar calculation may be done in the other models with random study intercepts. Estimating marginal estimands in models with fixed study intercepts is harder. We would need to perform a separate meta‐analysis to pool the underlying risk, and somehow combine the two meta‐analyses using integration over the heterogeneity terms $\delta_{ib_{i}k}^{c}$ or $\delta_{i1k}^{c}$. A simpler approach is to apply the estimated conditional odds ratio to the mean underlying risk, and this is useful to estimate quantities such as the risk difference, but care must be taken to fully allow for uncertainty and heterogeneity.

The marginal estimands discussed above use the average underlying risk of the studies in the NMA, which is unlikely to be representative of the target population. External information about clinical populations is therefore valuable for such an analysis. Dias and Ades^4^ argued that, while the overall intervention effect is best estimated in the NMA data set (because randomization promotes internal validity), the overall outcome prevalence is best estimated from clinical registries or other observational sources external to the NMA data set. Any of the models can be used in conjunction with external information to estimate the marginal effect of treatment in a well‐defined population.^4^

## 4 RESPECTING RANDOMISATION

One consequence of using random study intercepts is that the estimated study intercepts are shrunk toward the overall mean, and therefore, the treatment effect estimated within a study is influenced by information outside that study. In other words, the models allow the use of between‐study information. This conflicts with the “principle of concurrent control,” that treated individuals should only be compared with randomized controls.^16^ It can also be described as “breaking randomization.”^4^ Here, we call it *compromising* as opposed to *respecting randomization*. It is not clear whether compromising randomization is a problem in practice. Senn^16^ wrote “I consider that in practice little harm is likely to be done” and other authors have similarly found little bias (eg, see the work of Achana et al^22^). We explore the problem of compromising randomization using hypothetical data designed to produce bias.

### 4.1 Hypothetical NMA data sets

We construct 10 data sets for a network of three treatments X, Y, and Z, where all studies have an X arm, some studies compare Y with X, some studies compare Z with X, and no studies compare Z directly with Y. Treatments Y and Z are in fact identical, and we explore the estimated Z‐Y contrast using the various NMA models for a binary event representing a successful outcome.

**Figure 1:**
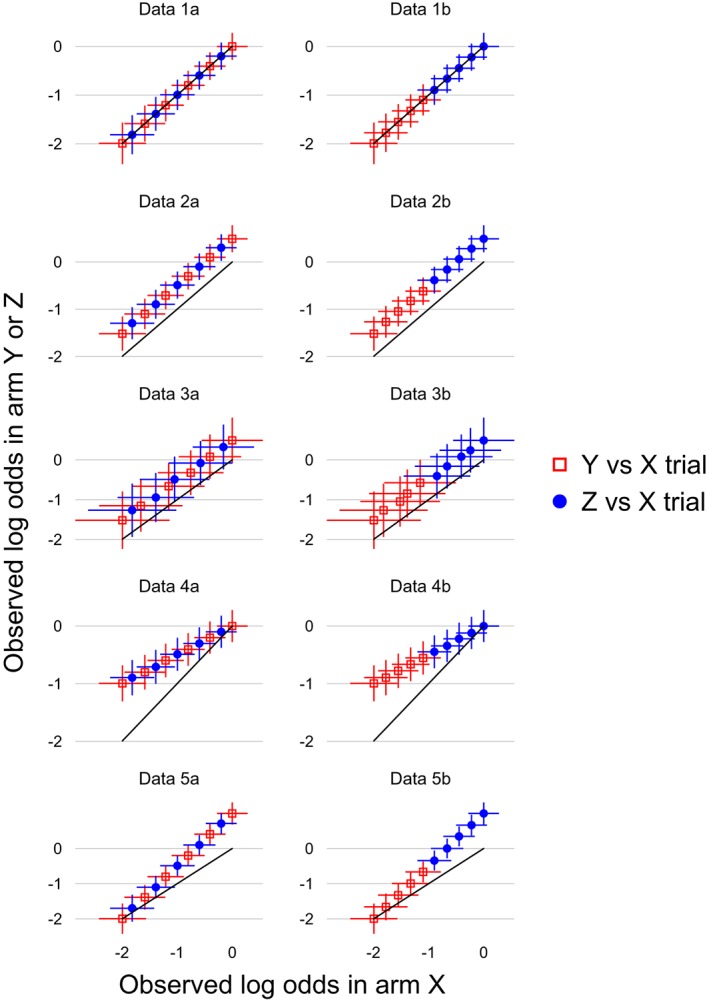
Hypothetical data sets used to compare models. The solid line indicates points with no treatment effect; points above this line have better outcomes in arm Y or Z than in arm X [Colour figure can be viewed at http://wileyonlinelibrary.com]

The 10 data sets are displayed in L'Abbé plots^23^ in Figure 1. They are described by a scenario (1 to 5) describing studies' treatment effects and sample sizes, and a data type (a/b) describing studies' choices of Y or Z (summarized in Table 1). Scenarios 1 to 3 assess the importance of respecting randomization and are introduced here; scenarios 4 and 5 assess missing data assumptions and are introduced in Section 5.3. The log odds of an event on treatment X varies systematically from −2 to 0 across studies, so that the overall event fraction is about 25%. In scenarios 1 and 2, each arm contains 200 patients. The log odds ratio (treatment effect) is 0 in all studies in scenario 1 and 0.5 in all studies in scenario 2. Scenario 3 is like scenario 2 but with the sample size reduced to 50 per arm.

**Table 1:** Summary of hypothetical data sets

Data scenario	Log odds ratio, Y/Z versus X	Sample size per arm	Data sub scenario	Designs
1	0 in all studies	200	a	Y versus X and Z versus X trials are similar
2	0.5 in all studies	200		
3	0.5 in all studies	50		
4	Average 0.5, decreasing with underlying risk	200	b	Y versus X trials have lower underlying risk,
5	Average 0.5, increasing with underlying risk	200		Z versus X trials have higher underlying risk

For each scenario, we create two data sets where the between‐studies information agrees (data type a) or disagrees (data type b) with the within‐studies information, so that analyses that use the between‐studies information, are likely to be biased only in data type b. In data type a, studies comparing X with Y are similar to studies comparing X with Z: this is approximated by interleaving a sequence of six Y versus X designs with five Z versus X designs. In data type b, five studies of Y versus X have low event fraction on X, and five studies of Z versus X have high event fraction on X.

The impact of between‐study information, if used, is that the estimated outcomes in the X arms of Y‐X studies are larger than observed in studies with low observed X outcome and smaller than observed in studies with high observed X outcome. In data type a, Y‐X studies have both low and high observed X outcome, so using between‐study information should not cause bias. In data type b, however, Y‐X studies have low observed X outcome, so the estimated outcomes in the X arms of Y‐X studies tend to be larger than observed, biasing the overall mean Y‐X contrast downwards; a similar argument suggests upwards bias in the overall Z‐X contrast, and hence larger upwards bias in the Z‐Y contrast.

For scenarios 1 to 3, the Y‐X and Z‐X contrasts have the same variance, while there is no evidence about the Z‐Y contrast. Thus models 2 and 3 hold with CH. Similarly, the arm‐specific variances are the same for all arms, so model 4 holds with CH. The models are summarized in Table 2.

**Table 2:** Summary of models

Model number	Description	Heterogeneity variance	Description
1	Contrast‐based model describing observed arms (Lu and Ades, 2006)	CH	Common heterogeneity
2	Contrast‐based model describing all possible arms	NCH	Noncommon heterogeneity
3	Contrast‐based model with random study intercepts		
4	Model with random study intercepts related to treatment effects (Hong et al, 2016)		

### 4.2 Results for hypothetical NMA data sets

**Figure 2:**
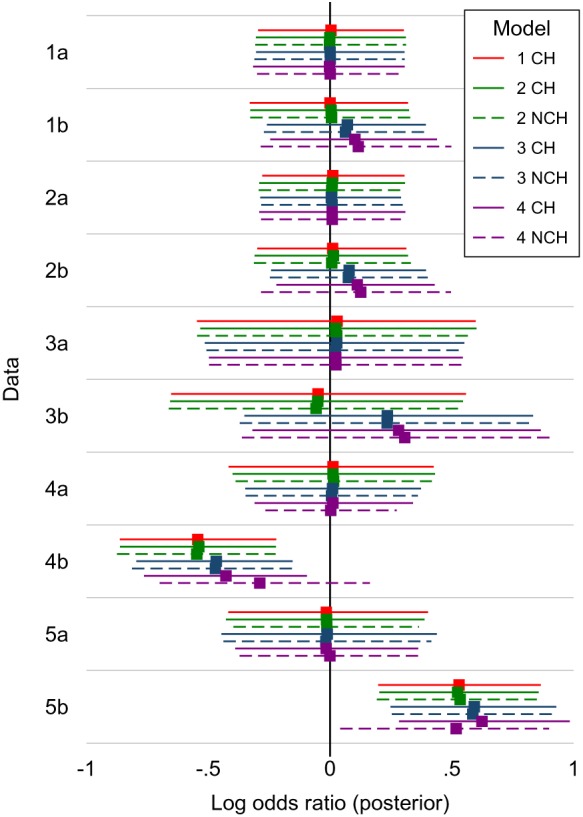
Analysis of hypothetical data sets: estimated treatment contrasts for Z versus Y, showing posterior median of the log odds ratio with 95% credible interval; vertical line shows the true value of zero. CH = common heterogeneity (solid lines); NCH = non‐common heterogeneity (dashed lines) [Colour figure can be viewed at http://wileyonlinelibrary.com]

The hypothetical data sets were analyzed using WinBUGS^24^ with a burn‐in of 50 000 updates and a further 200 000 updates, thinned to every 20th update. This yielded autocorrelations below 0.2 for all parameters at lag 2 for models 1 CH and 4 CH, at lag 4 for model 2 CH and 3 CH, at lag 6 for models 2 NCH and 3 NCH, and at lag 15 for model 4 NCH. Results for the overall mean treatment effect for Z versus Y are shown in the top six panels of Figure 2. Results for comparisons with X are in Supplementary Figure S1.

For data 1a, 2a, and 3a, where studies of the two designs are similar and hence there is no potential bias from between‐study information to be drawn, all models give results with posterior median close to the true value, with similar credible intervals.

For data 1b, 2b, and 3b, where studies of the two designs differ and hence there is potential bias from between‐study information, models 1 and 2 give results with posterior median close to the true value, while models 3 and 4 give posterior medians different from the true value. For data 1b and 2b, model 3 differs from the true value by 0.07 to 0.08, and model 4 differs by 0.10 to 0.12. For data 3b, where the studies were smaller, the degree of between‐study information was larger, with the Z‐Y contrast being estimated at 0.24 by model 3 and 0.29 to 0.30 by model 4. This corresponds to an odds ratio of 1.35 when the truth is an OR of 1. Thus, bias can be substantial in extreme cases.

Results for the contrast heterogeneity standard deviation are shown in Supplementary Figure S2 and for the arm heterogeneity standard deviation in Supplementary Figure S3. For each of data 1 to 3, all models have similar estimates of these parameters.

We also verified that the priors were similar between models by drawing 10 000 samples from the priors (Supplementary Figure S4). Priors for overall mean treatment effects were very flat. Priors for heterogeneity variances were broadly similar, but some differences were seen; although we have matched the prior means and spread of the log heterogeneity variances, some distributions were more positively skewed than others.

## 5 MISSING DATA

A key claim made for the AB approach is that it can “gain additional information from the incomplete records.”^3^ Here, we evaluate this claim from a missing data perspective, considering first the information in the missing data and then the assumptions made about the missing data. We take the missing data for each study to be the data for the treatment arms that were not included in that study. We assume that the sizes of these missing treatment arms are known, which is often reasonable, since most studies have equal sizes for all new treatment arms; we return to this issue in the discussion.

All the analyses considered here make a missing at random (MAR) assumption, because they are fitted to the observed data without modeling the mechanism by which those data come to be observed.^25^ The MAR assumption states that the probability of a particular design being chosen can depend only on the results for the treatments in that design; formally,

$$p(R_{i}=r|Y_{i})=p\left(R_{i}=r|Y_{i}^{obs(r)}\right),$$

where *R*_*i*_ is as before the design of study *i*, *Y*_*i*_ is the complete data in study *i*, and $Y_{i}^{obs(r)}$ is the part of *Y*_*i*_ containing the results for the treatments that are observed if *R*_*i*_=*r*. The implications of MAR depend on two model aspects, which we discuss below: whether we use a CB likelihood or an AB likelihood (hence, whether *Y*_*i*_ is the set of contrasts or the set of arm summaries), and whether the model is correctly specified.

### 5.1 Nature of MAR assumptions

For a CB likelihood, the MAR assumption is that the probability of a particular design being chosen does not depend on the unobserved *contrasts*, given the observed contrasts. We call this the “C‐MAR” assumption. For an AB likelihood, however, the MAR assumption is that the probability of a particular design being chosen does not depend on the unobserved arm *counts*, given the observed arm counts. We call this the “A‐MAR” assumption. Formal definitions are given in Supplementary Appendix F.

We next consider what happens if the AB likelihood is misspecified. Models 1 to 3 assume that the outcomes in arm 1 and the contrasts with arm 1 are independent. If arm 1 is always observed, the likelihood factorizes into the likelihood for arm 1 and the likelihood for the contrasts. In this case, the validity of inference about the contrasts clearly depends on the “C‐MAR” assumption and not on the “A‐MAR assumption.” Even if arm 1 is not always observed, a similar result seems likely to hold. Thus, under A‐MAR and subject to distributional assumptions, likelihood‐based techniques validly estimate model 4, but are unlikely to validly estimate models 1 to 3. We explore this below.

It is sometimes claimed that NMA with a CB likelihood assumes that the data are missing completely at random.^26^ In Supplementary Appendix G, we show that this is only true in special cases and that often the required assumption is weaker than missing completely at random.

### 5.2 When do the differences between C‐MAR and A‐MAR matter?

The key difference between C‐MAR and A‐MAR is that A‐MAR holds even when choice of design depends on the arm‐specific means and not just the contrasts. As an example of data that may be A‐MAR but not C‐MAR, consider the case where all studies include arm X, and studies of more seriously ill patients (with higher event rate) tend to compare X with Y, while studies of less seriously ill patients (with lower event rate) tend to compare X with Z. These data may be A‐MAR because choice of design depends on data that are observed and included (actual outcome in arm X). Whether they are C‐MAR depends on whether ignoring actual outcome in arm X induces a relationship between choice of design and the actual contrasts. This happens if the outcome in arm X is related both to the choice of comparator Y or Z (design) and to the Y‐X and Z‐X contrasts. In this case, underlying risk is an effect modifier, which differs systematically between the X‐Y studies and X‐Z studies. This violates the idea of transitivity, which may be stated as “sets of trials do not differ with respect to the distribution of effect modifiers”^27^; in this case, NMA reviewers are explicitly told not to use indirect comparisons.

In summary, it seems that models 1 to 3 may suffer from missing data bias when data are A‐MAR and the outcome in the reference arm is associated both with study design and with treatment contrasts, while model 4 may be able to handle this form of violation of transitivity.

### 5.3 Exploration in hypothetical data

We use two further scenarios where treatment effects are negatively associated (scenario 4) and positively associated (scenario 5) with arm X risk (Figure 1). In both cases, the data are designed so that the average log odds is −1 on arm X and −0.5 on arms Y and Z. Here, the CH assumption is true for models 1 to 3 (heterogeneity is the same for Y‐X and Z‐X contrasts) but not for model 4 (heterogeneity for arm X differs from that for arms Y and Z). The C‐MAR and A‐MAR assumptions are both true for data 1 to 3, 4a, and 5a. However, for data 4b and 5b, A‐MAR is true and C‐MAR is false.

Results are shown in Figure 2. Data 4a and 5a show the expected correct results for all models. Data 4b and 5b show the expected missing data bias, ie, estimates are in error by around −0.5 in data 4b, and around +0.5 in data 5b. Model 4 with NCH goes some way toward correcting this missing data bias. Results for data 4b are encouraging because the two phenomena (using between‐study information and correcting missing‐data bias) both move the point estimate towards the true value. However this is not necessarily the case, and results for data 5b show the two effects cancelling out; consequently, model 4 gives results similar to those from models 1 and 2.

Overall, these results are not encouraging for model 4. When it reduced bias (data 4b), it only removed half the bias (and only in the NCH case), and part of the bias reduction arose from a second bias (from compromising randomization) acting in the opposite direction.

## 6 EXAMPLE: INHALED CORTICOSTEROIDS

From an ongoing empirical investigation,^28^ we select one network, which shows a large difference between models, in order to improve our understanding of the differences between the models. The selected network comprises 18 randomized trials comparing seven inhaled corticosteroids with placebo in the treatment of chronic asthma.^29^ The treatments are coded: 1, placebo; 2, beclomethasone; 3, budesonide; 4, ciclesonide; 5, flunisolide; 6, fluticasone; 7, mometasone; and 8, triamcinolone. The outcome considered here is elimination of oral corticosteroid use. The data (Table 3) have zero events in four of the control arms, which is handled by our use of the exact binomial likelihood for the data.

**Table 3:** Inhaled corticosteroids network: data

Study	Active arm			Placebo arm		
	Treatment	Events	Patients	Treatment	Events	Patients
1	7	27	84	1	1	38
2	7	34	88	1	0	43
3	3	62	88	1	4	51
4	3	12	65	1	1	30
5	2	48	61	1	21	54
6	2	10	17	1	2	16
7	2	13	15	1	1	12
8	2	18	68	1	1	33
9	2	10	11	1	0	11
10	2	11	13	1	0	12
11	2	2	10	1	0	10
12	6	62	173	1	16	78
13	6	62	76	1	3	33
14	6	50	62	1	1	30
15	8	13	16	1	3	17
16	4	29	92	1	5	44
17	5	11	40	1	4	33
18	5	2	17	1	1	15

**Figure 3:**
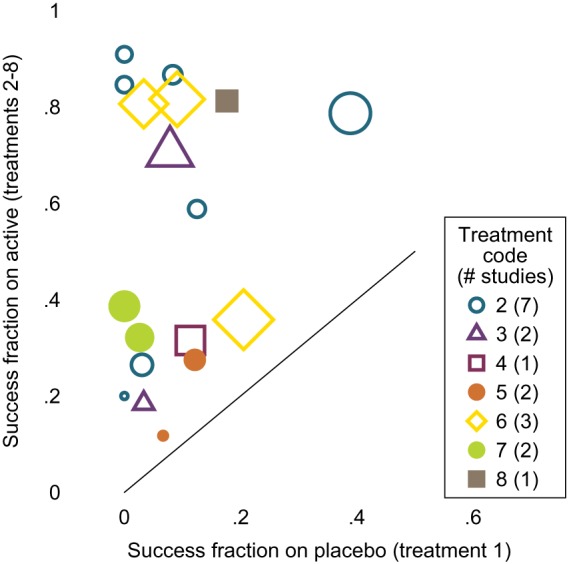
Inhaled corticosteroids network: L'Abbé plot. Symbol size is proportional to number of events in study [Colour figure can be viewed at http://wileyonlinelibrary.com]

**Figure 4:**
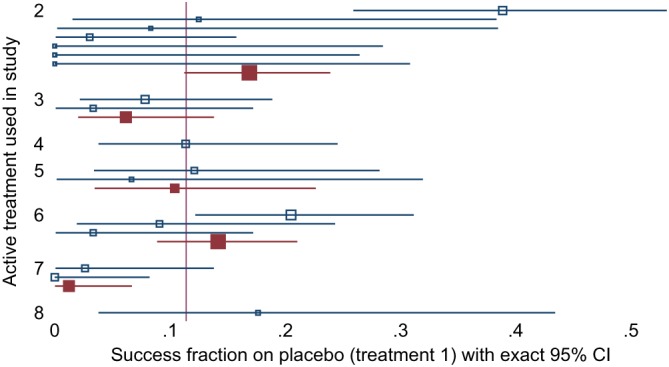
Inhaled corticosteroids network: forest plot showing success fraction on placebo arms for each study (open symbols) and summarizing studies with the same comparator (filled symbols; only shown where more than one study has the same comparator; studies weighted by sample size). Some placebo arms have no events, so exact confidence intervals are shown for all studies. Symbol size is proportional to number of individuals in the placebo arm. CI, confidence interval [Colour figure can be viewed at http://wileyonlinelibrary.com]

There are no head‐to‐head comparisons of the inhaled corticosteroids so this is a “star” network (Supplementary Figure S5). A L'Abbé plot shows that the studies vary widely both in treatment effect (distance from the diagonal line) and underlying risk (horizontal axis) (Figure 3). Variation in underlying risk is further explored in Figure 4. Underlying risk is very low for studies of treatment 7, low for studies of treatment 3, and highest for studies of treatments 2 and 8. However, there is large heterogeneity between studies with the same comparator, and some studies of treatment 2 have very low underlying risk. Because of the small numbers of studies for most contrasts, we fit only CH models to these data. Numbers of updates were as in Section 4.2, and all autocorrelations were below 0.2 by lag 8 (after thinning).

**Figure 5:**
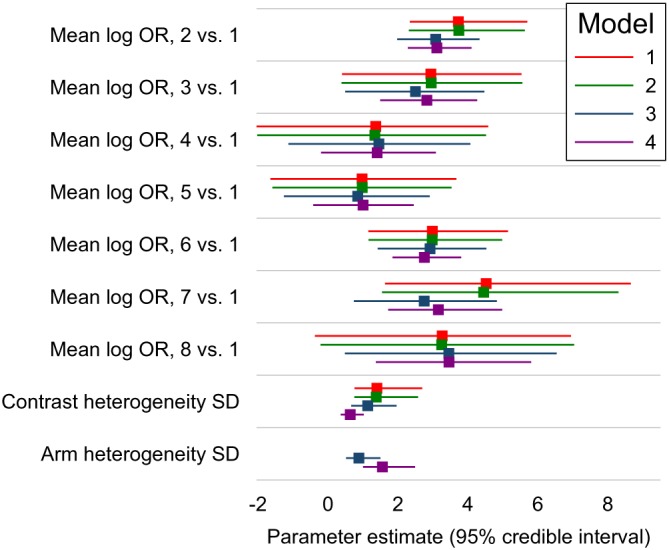
Inhaled corticosteroids network: results of Bayesian analyses with common heterogeneity models, showing posterior median with 95% credible interval for the mean treatment contrasts (log odds ratios, OR) for treatments 2 to 7 compared with treatment 1 ( $\mu_{1k}^{c}$), the contrast heterogeneity standard deviation (SD) (*σ* ^*c*^ in all models), and the arm heterogeneity SD (*σ* ^*a*^ in models 3 and 4) [Colour figure can be viewed at http://wileyonlinelibrary.com]

Results of fitting all models are shown in Figure 5. Models 1 and 2 give very similar results. Compared with models 1 and 2, model 3 gives estimated overall mean treatment effects for treatment 7 versus 1 that are 2 units smaller, corresponding to odds ratios seven to eight times smaller, a huge discrepancy. Smaller discrepancies are seen for contrasts 2 versus 1 (0.7 units smaller), 3 versus 1 (0.5 units smaller), and 8 versus 1 (0.3 units larger). Results for other overall mean treatment effects compared with treatment 1 differed by less than 0.1. The low result for treatment 7 is easily explained by the very low underlying risk in studies of treatment 7, noted above: the between‐studies information in model 3 shrinks the underlying risk upwards in these studies and hence reduces the treatment effect. The less dramatic results for treatments 3 and 8 have similar explanations. The result for treatment 2 is harder to explain, given its high underlying risk: it may be that the small studies of treatment 2 with zero estimated underlying risk (Figure 4) receive greater weight in the model fitting (which allows for heterogeneity) than in the pooled calculation of underlying risk (which weighted studies by sample size), and this may decrease the effective underlying risk in studies of treatment 2.

Model 4 gives results similar to model 3 in most cases, and somewhat closer to models 1 and 2 in some cases, with no difference larger than 0.3 (for treatment 7). One possible explanation for differences between models 4 and 3 is that the treatment effect may relate to underlying risk (Section 5). However, results from model 4 suggest little association between treatment effect and underlying risk, with the parameter *ρ* ^*a*^ in model (12) estimated close to 1 (0.85 with 95% credible interval 0.72 to 0.94). A more likely explanation lies in the estimate of *σ* ^*a*^, which is about 0.5 units larger in model 4 than in model 3 (Figure 5). The treatment effect of 7 versus 1 is strongly associated with *σ* ^*a*^, with a regression slope of 0.7 in the posterior for model 4 (results not shown): together, these explain the treatment effect increase in model 4.

## 7 DISCUSSION

### 7.1 Main messages

We set out to compare CB and AB models. We found that AB models are mathematically neater, but otherwise very similar to certain CB models. The important differences lie not between CB and AB models, but between other model features.

There has been debate between one specific CB model (model 1) and one specific AB model (model 4).^3^, ^4^ The differences between these models are much greater than between CB and AB models in general, and we used intermediate models 2 and 3 to clarify them. Key differences, summarized in Table 4, were fixed versus random study intercepts, estimands, whether treatment effects relate to study intercepts, and missing data assumptions.

**Table 4:** Summary of model properties, assuming analysis on the odds ratio (OR) scale

Model	Main estimand	Other estimands	Between‐study information	Missing data assumption
1 and 2	Conditional OR	Any marginal, given external data	Not used	C‐MAR
3	Conditional OR	Any marginal, given external data	Used	C‐MAR
4	Conditional or marginal OR	Any marginal, given external data	Used	A‐MAR

Compromising randomization through the use of random study intercepts is a feature of models 3 and 4. We found that this can introduce important bias (Sections 4 and 6). More research is needed to identify any situations where this could be of practical importance, but it must be a concern. Random study intercepts are however useful in solving otherwise impossible problems such as disconnected networks.^30^

All models naturally estimate an average study‐specific treatment effect such as an odds ratio. Other estimands may be of interest when (as is usually the case) studies vary in their underlying risk (eg, their control group success fractions). Models with random study intercepts (as in models 3 and 4) facilitate estimation of a marginal treatment effect, such as an absolute risk reduction, across the population of studies. However, often external data are used to estimate the underlying risk in a target population, and then the absolute risk reduction in the target population can be estimated from any of the models (Section 3).

Model 4 allows the treatment effect to vary with underlying risk. It therefore estimates the treatment effect at the average level of underlying outcomes, where the average is over the studies in the NMA. If there were evidence that treatment effect varied with underlying risk, then the data analyst would replace these overall summaries with summaries at specific levels of underlying risk. In the absence of such evidence, however, it still seems likely that treatment effect may vary with underlying risk, and in this case, it is not clear what the estimand is for models 1 to 3. Models allowing the treatment effect to vary with underlying risk have been described previously^22^; unfortunately, models that respect randomization by using fixed study intercepts may lead to inconsistent likelihood‐based estimation.^31^

Likelihood‐based estimation of CB and AB models under ignorability make different MAR assumptions, C‐MAR, and A‐MAR (Section 5). The AB models are theoretically likely to give better answers when underlying risk is related both to treatment effect and to study design, but we did not find empirical evidence of this.

We also found that choice of prior is not simple in models with NCH, since standard choices of inverse Wishart priors tend to be quite informative. Choice of prior is also not simple in model 4 with CH, since this involves two different quantities (arm heterogeneity and contrast heterogeneity) which need separate priors. However, we were able to derive evidence‐based priors and use them systematically across models, so that priors were similar across models (Section 2.4).

### 7.2 Limitations

We defined the complete data as the results for all possible treatment arms, assuming known sample sizes (Section 5). We could alternatively consider the sample sizes of the missing arms (1) as missing data or (2) as zero. In case (1), we would need an additional statistical model for the sample sizes. In case (2), there are no missing data and instead we must model the observation process. The problem of arm sizes is closely bound up with that of missing data; for example, we have discussed how bias would arise if a study arm were included or excluded dependent on the results expected in that study arm, but bias would also arise if a study arm's size was chosen dependent on the results expected in that study arm. Methods for informative cluster size^32^ may be useful here.

Zhang et al also tackled missing data issues by implicitly considering the missing arm sizes as known.^26^ They considered missing not at random (MNAR) selection models, which allow the probabilities of a particular arm being observed to depend on the event fraction in that arm. In practice, study design occurs before patient recruitment and therefore is most unlikely to depend on the actual results that would be observed if a particular design was adopted. Instead, study design is likely to depend on the underlying parameters $\theta_{ik}^{a}$. It would be interesting to see future work that relates study design, size, and allocation ratio to these underlying parameters, ie, that relates *R*_*i*_ and {*n*_*ik*_:*k*∈*R*_*i*_} to $\left\{\theta_{ik}^{a}:k=1,\text{…},K\right\}$.

Our analysis focused on specific hypothetical and real data sets. We have only considered star‐shaped networks. We have not repeatedly drawn data from specific models, so we are unable to systematically compare results across models or to evaluate standard errors and confidence intervals. An empirical comparison is under way^28^ and future research should assess the performance of these models using simulation studies.

The models proposed apply for AB likelihoods. With a CB likelihood, the study intercepts are implicitly treated as fixed effects, so models 3 and 4 are not possible.

### 7.3 Extensions

We regarded the mean treatment effects as separate parameters. However, it is sometimes possible to gain precision through modeling assumptions on these parameters. Where some treatments are different doses of the same drug, modeling assumptions may be made across doses.^33^ Where some treatments are drugs in the same class, related treatments could be allowed to have related effects.^34^ Where treatments are combinations of component treatments, as in complex interventions, overall effects could be modeled in terms of the effects of the intervention components (and possibly interactions).^35^ These models for the treatment effects could be combined with any of our models.

We have assumed consistency. In the presence of inconsistency, heterogeneity parameters such as *σ* ^*c*^ represent heterogeneity plus inconsistency. Any of the models in this paper could be extended to include inconsistency terms.^10^, ^18^

The models in this paper apply to other metrics for binary data, including the risk ratio and risk difference, and to other data types, such as count and continuous data, by changing the measurement model; the structural models are unchanged. Marginal and conditional estimands are similar with continuous data. Binary, count, and continuous data have the advantage that an exact AB likelihood can be constructed from aggregate data. For time‐to‐event data, aggregate data allow only a CB likelihood, which allows only models 1 and 2 to be fitted. Individual participant data with a time‐to‐event outcome allow all models to be fitted, but Bayesian estimation of such NMA models is complex.^36^

Jackson et al recently explored seven models for frequentist analysis of pairwise meta‐analysis using AB likelihoods.^12^ Two models had fixed study intercepts and coded treatment as 0/1 (model 2) and −0.5/0.5 (model 4), and substantial underestimation of the heterogeneity variance was found for model 2 but not for model 4. Three models (3, 5, and 6) had random study intercepts and performed well, with only minor bias when between‐study information disagreed with randomized information. The models in the present paper can also be applied to pairwise meta‐analysis; in this case, our models 1 to 4 reduce to Jackson et al's models 2, 2, 3, and 6, respectively. The AB expression of our model 2 does not reduce to any of Jackson et al's models and has been shown to differ subtly from our model 1 in the frequentist setting.^8^, ^15^ However, the biased estimation of the heterogeneity variance found in frequentist analysis does not appear to extend to the Bayesian estimation used in this paper. In fact, the methods of supplementary Appendix A can be used to show that Jackson et al's models 2 and 4 are equivalent under Bayesian estimation with flat priors for the study intercepts. Future research should explore performance of the different models in a Bayesian setting and explore NMA equivalents of Jackson et al's models 4 and 5.

Handling multiarm studies is straightforward in models 2 to 4 and has been described for model 1.^37^ Finally, we have assumed normal distributions for the random study intercepts, but this can be relaxed to a mixture of normals.^38^

### 7.4 Conclusions

The most important difference between models is not whether they are CB or AB, but whether they have random study intercepts. Models with random study intercepts have both appealing and unappealing properties, but their main weakness is susceptibility to bias when there are systematic differences between trials of different designs, and the evidence does not at present support their routine use. Models with fixed study intercepts can be recommended and may be implemented with either a CB or an AB model.

## Supporting information

SIM_8360‐Supp‐0001‐ABCB_supplementary.pdf

SIM_8360‐Supp‐0002‐ABCB_datasharing.zip

## References

[1] Petropoulou M , Nikolakopoulou A , Veroniki AA , et al. Bibliographic study showed improving statistical methodology of network meta‐analyses published between 1999 and 2015. J Clin Epidemiol. 2017;82:20‐28. 27864068 10.1016/j.jclinepi.2016.11.002

[2] Zhang J , Carlin BP , Neaton JD , et al. Network meta‐analysis of randomized clinical trials: reporting the proper summaries. Clinical Trials. 2014;11(2):246‐262. 24096635 10.1177/1740774513498322 PMC3972291

[3] Hong H , Chu H , Zhang J , Carlin BP . A Bayesian missing data framework for generalized multiple outcome mixed treatment comparisons. Res Synth Methods. 2016;7(1):6‐22. 26536149 10.1002/jrsm.1153 PMC4779385

[4] Dias S , Ades AE . Absolute or relative effects? Arm‐based synthesis of trial data. Res Synth Methods. 2016;7(1):23‐28. 26461457 10.1002/jrsm.1184 PMC5102631

[5] Hong H , Chu H , Zhang J , Carlin BP . Rejoinder to the discussion of “A Bayesian missing data framework for generalized multiple outcome mixed treatment comparisons”, by S. Dias and A.E. Ades. Res Synth Methods. 2016;7(1):29‐33. 26461816 10.1002/jrsm.1186 PMC4779393

[6] Salanti G , Higgins JPT , Ades AE , Ioannidis JPA . Evaluation of networks of randomized trials. Stat Methods Med Res. 2008;17:279‐301. 17925316 10.1177/0962280207080643

[7] Hawkins N , Scott DA , Woods B . ‘Arm‐based’ parameterization for network meta‐analysis. Res Synth Methods. 2016;7(3):306‐313. 26610409 10.1002/jrsm.1187 PMC5063191

[8] Piepho HP , Madden LV , Roger J , Payne R , Williams ER . Estimating the variance for heterogeneity in arm‐based network meta‐analysis. Pharmaceutical Statistics. 2018;17(3):264‐277. 29676023 10.1002/pst.1857

[9] Higgins JPT , Thompson SG , Spiegelhalter DJ . A re‐evaluation of random‐effects meta‐analysis. J Royal Stat Soc: Ser A (Stat Soc). 2009;172(1):137‐159. 10.1111/j.1467-985X.2008.00552.x PMC2667312 19381330

[10] Higgins JPT , Jackson D , Barrett JL , Lu G , Ades AE , White IR . Consistency and inconsistency in network meta‐analysis: concepts and models for multi‐arm studies. Res Synth Methods. 2012;3:98‐110. 26062084 10.1002/jrsm.1044 PMC4433772

[11] Bowden J , Tierney JF , Simmonds M , Copas A , Higgins JP . Individual patient data meta‐analysis of time‐to‐event outcomes: one‐stage versus two‐stage approaches for estimating the hazard ratio under a random effects model. Res Synth Methods. 2011;2(3):150‐162. 26061783 10.1002/jrsm.45

[12] Jackson D , Law M , Stijnen T , Viechtbauer W , White IR . A comparison of seven random‐effects models for meta‐analyses that estimate the summary odds ratio. Statist Med. 2018;37(7):1059‐1085. 10.1002/sim.7588 PMC5841569 29315733

[13] Lu G , Ades AE . Assessing evidence inconsistency in mixed treatment comparisons. J Am Stat Assoc. 2006;101:447‐459.

[14] Thompson SG , Smith TC , Sharp SJ . Investigating underlying risk as a source of heterogeneity in meta‐analysis. Statist Med. 1997;16(23):2741‐2758. 10.1002/(sici)1097-0258(19971215)16:23<2741::aid-sim703>3.0.co;2-0 9421873

[15] Piepho HP , Williams ER , Madden LV . The use of two‐way linear mixed models in multitreatment meta‐analysis. Biometrics. 2012;68(4):1269‐1277. 22845838 10.1111/j.1541-0420.2012.01786.x

[16] Senn S . Hans van Houwelingen and the art of summing up. Biometrical Journal. 2010;52(1):85‐94. 20140900 10.1002/bimj.200900074

[17] Lu G , Ades AE . Modeling between‐trial variance structure in mixed treatment comparisons. Biostatistics. 2009;10:792‐805. 19687150 10.1093/biostatistics/kxp032

[18] White IR , Barrett JK , Jackson D , Higgins JPT . Consistency and inconsistency in network meta‐analysis: model estimation using multivariate meta‐regression. Res Synth Methods. 2012;3(2):111‐125. 26062085 10.1002/jrsm.1045 PMC4433771

[19] Thorlund K , Thabane L , Mills E . Modelling heterogeneity variances in multiple treatment comparison meta‐analysis–are informative priors the better solution? BMC Med Res Methodol. 2013;13:2. 23311298 10.1186/1471-2288-13-2 PMC3554418

[20] Turner RM , Davey J , Clarke MJ , Thompson SG , Higgins JPT . Predicting the extent of heterogeneity in meta‐analysis, using empirical data from the Cochrane database of systematic reviews. Int J Epidemiol. 2012;41(3):818‐827. 22461129 10.1093/ije/dys041 PMC3396310

[21] Neuhaus JM , Kalbfleisch JD , Hauck WW . A comparison of cluster‐specific and population‐averaged approaches for analyzing correlated binary data. Int Stat Rev/Revue Int Stat. 1991;59(1):25‐35.

[22] Achana FA , Cooper NJ , Dias S , et al. Extending methods for investigating the relationship between treatment effect and baseline risk from pairwise meta‐analysis to network meta‐analysis. Statist Med. 2013;32(5):752‐771. 10.1002/sim.5539 22865748

[23] L'Abbé KA , Detsky AS , O'rourke K . Meta‐analysis in clinical research. Ann Intern Med. 1987;107(2):224‐233. 3300460 10.7326/0003-4819-107-2-224

[24] Spiegelhalter D , Thomas A , Best N , Lunn D . WinBUGS User Manual Version 1.4. 2003.

[25] Little RJA , Rubin DB . Statistical Analysis With Missing Data. 2nd ed. Hoboken, NJ: Wiley; 2002.

[26] Zhang J , Chu H , Hong H , Virnig BA , Carlin BP . Bayesian hierarchical models for network meta‐analysis incorporating nonignorable missingness. Stat Methods Med Res. 2017;26(5):2227‐2243. 26220535 10.1177/0962280215596185 PMC4731325

[27] Salanti G . Indirect and mixed‐treatment comparison, network, or multiple‐treatments meta‐analysis: many names, many benefits, many concerns for the next generation evidence synthesis tool. Res Synth Methods. 2012;3:80‐97. 26062083 10.1002/jrsm.1037

[28] Karahalios AA , Salanti G , Turner SL , et al. An investigation of the impact of using different methods for network meta‐analysis: a protocol for an empirical evaluation. Systematic Reviews. 2017;6(1):119. 28646922 10.1186/s13643-017-0511-x PMC5483272

[29] Abdullah AK , Khan S . Relative oral corticosteroid‐sparing effect of 7 inhaled corticosteroids in chronic asthma: a meta‐analysis. Ann Allergy Asthma Immunol. 2008;101(1):74‐81. 18681088 10.1016/S1081-1206(10)60838-1

[30] Béliveau A , Goring S , Platt RW , Gustafson P . Network meta‐analysis of disconnected networks: how dangerous are random baseline treatment effects? Res Synth Methods. 2017;8(4):465‐474. 28737842 10.1002/jrsm.1256

[31] van Houwelingen HC , Arends LR , Stijnen T . Advanced methods in meta‐analysis: multivariate approach and meta‐regression. Statist Med. 2002;21(4):589‐624. 10.1002/sim.1040 11836738

[32] Seaman S , Pavlou M , Copas A . Review of methods for handling confounding by cluster and informative cluster size in clustered data. Statist Med. 2014;33(30):5371‐5387. 10.1002/sim.6277 PMC4320764 25087978

[33] Giovane CD , Vacchi L , Mavridis D , Filippini G , Salanti G . Network meta‐analysis models to account for variability in treatment definitions: application to dose effects. Statist Med. 2013;32(1):25‐39. 10.1002/sim.5512 22815277

[34] Owen RK , Tincello DG , Abrams KR . Network meta‐analysis: development of a three‐level hierarchical modeling approach incorporating dose‐related constraints. Value Health. 2015;18(1):116‐126. 25595242 10.1016/j.jval.2014.10.006

[35] Welton NJ , Caldwell DM , Adamopoulos E , Vedhara K . Mixed treatment comparison meta‐analysis of complex interventions: psychological interventions in coronary heart disease. Am J Epidemiol. 2009;169(9):1158‐1165. 19258485 10.1093/aje/kwp014

[36] Freeman SC . *One‐Step Individual Participant Data Network Meta‐Analysis of Time‐to‐Event Data* [PhD thesis]. London, UK: University College London; 2016.

[37] Dias S , Sutton AJ , Ades AE , Welton NJ . A generalized linear modeling framework for pairwise and network meta‐analysis of randomized controlled trials. Med Decis Mak. 2013;33:607‐617. 10.1177/0272989X12458724 PMC3704203 23104435

[38] Arends LR , Hoes AW , Lubsen J , Grobbee DE , Stijnen T . Baseline risk as predictor of treatment benefit: three clinical meta‐re‐analyses. Statist Med. 2000;19(24):3497‐3518. 10.1002/1097-0258(20001230)19:24<3497::aid-sim830>3.0.co;2-h 11122510

