# Supplementary material for: A comparison of arm‐based and contrast‐based models for network meta‐analysis
Source: Stat Med. 2019 Oct 3;38(27):5197–213. doi: 10.1002/sim.8360 (PMC6899819; doi:10.1002/sim.8360)

Supplementary Materials

A Equivalence of model 1 and the CB and AB forms of model 2

We show that model 1 and the CB and AB forms of model 2 (here called 2CB and 2AB) in Section 2.3 are equivalent in a Bayesian model with flat priors on the study intercepts.

Since we do not consider a NCH version of model 1, we present this proof only for the CH model; similar methods can be used to prove equivalence of NCH versions of models 2CB and 2AB. For simplicity we consider two-arm trials not containing the reference treatment; similar methods can be used to prove equivalence in other cases.

Consider trial i comparing treatments b_i and k, where $1 < b_i < k$. Let $\boldsymbol{\theta}_i^a = (\theta_{ib_i}^a, \theta_{ik}^a)$. Let $\boldsymbol{\Phi}$ be the generic mean and variance parameters: for model 1, $\boldsymbol{\Phi} = (\{\mu_{1k}^c : k = 2, ..., K\}, \sigma^{c2})$; for model 2CB and 2AB, $\boldsymbol{\Phi} = (\boldsymbol{\mu}^c, \sigma^{c2})$. Let α_i^* be the generic intercept parameter for study i: for model 1, $\alpha_i^* = \alpha_{iB}^a$ $\alpha_i^* = \alpha_{ib_i}^a$; for model 2CB, $\alpha_i^* = \alpha_{i1}^a$; for model 2AB, $\alpha_i^* = \alpha_i^a$. Let these generic intercept parameters have $N(0, L)$ priors with large L.

Our approach is to show that $p(\boldsymbol{\theta}_i^a|\boldsymbol{\Phi})$ is the same for all models. This is sufficient if studies are independent given $\boldsymbol{\Phi}$, because

$$\begin{aligned} p(\boldsymbol{\Phi}|\boldsymbol{y}) &\propto p(\boldsymbol{\Phi}) \prod_i \iint p(\boldsymbol{y}_i|\boldsymbol{\theta}_i^a) p(\boldsymbol{\theta}_i^a|\alpha_i^*, \boldsymbol{\Phi}) p(\alpha_i^*|\boldsymbol{\Phi}) d\alpha_i^* d\boldsymbol{\theta}_i^a \\ &= p(\boldsymbol{\Phi}) \prod_i \iint p(\boldsymbol{y}_i|\boldsymbol{\theta}_i^a) p(\boldsymbol{\theta}_i^a|\boldsymbol{\Phi}) d\boldsymbol{\theta}_i^a \end{aligned}$$

and on the right hand side, $p(\boldsymbol{\Phi})$ and $p(\boldsymbol{y}_i|\boldsymbol{\theta}_i^a)$ are the same for all models.

Under model 1, we have

$$\begin{aligned} \boldsymbol{\theta}_i^a|\alpha_i^*, \boldsymbol{\Phi} &\sim N\left(\begin{pmatrix} \alpha_{ib_i}^a \\ \alpha_{ib_i}^a + \mu_{1k}^c - \mu_{1b_i}^c \end{pmatrix}, \begin{pmatrix} 0 & 0 \\ 0 & \sigma^{c2} \end{pmatrix}\right) \\ \text{and so } \boldsymbol{\theta}_i^a|\boldsymbol{\Phi} &\sim N\left(\begin{pmatrix} 0 \\ \mu_{1k}^c - \mu_{1b_i}^c \end{pmatrix}, \begin{pmatrix} L & L \\ L & L + \sigma^{c2} \end{pmatrix}\right). \end{aligned}$$

Under model 2CB, we have

$$\begin{aligned} \boldsymbol{\theta}_i^a|\alpha_i^*, \boldsymbol{\Phi} &\sim N\left(\begin{pmatrix} \alpha_{i1}^a + \mu_{b_i}^c \\ \alpha_{i1}^a + \mu_k^c \end{pmatrix}, \begin{pmatrix} \sigma^{c2} & \sigma^{c2}/2 \\ \sigma^{c2}/2 & \sigma^{c2} \end{pmatrix}\right) \\ \text{and so } \boldsymbol{\theta}_i^a|\boldsymbol{\Phi} &\sim N\left(\begin{pmatrix} \mu_{b_i}^c \\ \mu_k^c \end{pmatrix}, \begin{pmatrix} L + \sigma^{c2} & L + \sigma^{c2}/2 \\ L + \sigma^{c2}/2 & L + \sigma^{c2} \end{pmatrix}\right). \end{aligned}$$

Under model 2AB, we have

$$\begin{aligned} \boldsymbol{\theta}_i^a|\alpha_i^*, \boldsymbol{\Phi} &\sim N\left(\left(\begin{array}{c} \alpha_i^a + \mu_{b_i}^c \\ \alpha_i^a + \mu_k^c \end{array}\right), \left(\begin{array}{cc} \sigma^{c2}/2 & 0 \\ 0 & \sigma^{c2}/2 \end{array}\right)\right) \\ \text{and so } \boldsymbol{\theta}_i^a|\boldsymbol{\Phi} &\sim N\left(\left(\begin{array}{c} \mu_{b_i}^c \\ \mu_k^c \end{array}\right), \left(\begin{array}{cc} L+\sigma^{c2}/2 & L \\ L & L+\sigma^{c2}/2 \end{array}\right)\right). \end{aligned}$$

Clearly these are not the same distributions. However, we show that they are *effectively* the same distribution in the flat prior case. To do this we show for any 2-vector $\boldsymbol{w}$ that $\boldsymbol{w}'\boldsymbol{\theta}_i^a$ either has the same distribution under all three models, or it has large variance and hence a flat distribution under all three models. We consider two cases:

1. Case $\boldsymbol{w}' = (-v, v)'$ for some scalar v. In this case, $\boldsymbol{w}'\boldsymbol{\theta}_i^a$ is a multiple v of the contrast between treatments. Its distribution given $\boldsymbol{\Phi}$ is $N\left(v(\mu_{1k}^c - \mu_{1b_i}^c), v^2\sigma^{c2}\right)$ under model 1 and $N\left(v(\mu_k^c - \mu_{b_i}^c), v^2\sigma^{c2}\right)$ under models 2CB and 2AB. Setting $\mu_{1b_i}^c = \mu_{b_i}^c$ and $\mu_{1k}^c = \mu_k^c$ makes these identical.

2. Case $\boldsymbol{w}' \neq (-v, v)'$. In this case, $\boldsymbol{w}'\boldsymbol{\theta}_i^a$ is not a contrast between treatments, and it has large variance under each model. Thus the three distributions become effectively the same as the priors become flatter, that is, as L becomes large.

Hence the models are the same.

B Equivalence of CB and AB forms of model 4

We show that CB and AB forms of model 4 in Section 2.3 are equivalent by defining the transformation from the parameters $\boldsymbol{\mu}^*$ and $\boldsymbol{\Sigma}^*$ in equation (8) to the parameters $\boldsymbol{\mu}^a = (\mu_1^a, \ldots, \mu_K^a)^T$ and $\boldsymbol{\Sigma}^a$ in equation (9). Define the $K \times K$ matrix

$$\boldsymbol{M} = \left(\begin{array}{ccccc} 1 & 0 & 0 & \ldots & 0 \\ -1 & 1 & 0 & \ldots & 0 \\ -1 & 0 & 1 & \ldots & 0 \\ \vdots & \vdots & \vdots & \ddots & \vdots \\ -1 & 0 & 0 & \ldots & 1 \end{array}\right).$$

Then $\boldsymbol{\mu}^* = \boldsymbol{M}\boldsymbol{\mu}^a$ and $\boldsymbol{\Sigma}^* = \boldsymbol{M}\boldsymbol{\Sigma}^a\boldsymbol{M}^T$. $\boldsymbol{M}$ is invertible so we can also write $\boldsymbol{\mu}^a = \boldsymbol{M}^{-1}\boldsymbol{\mu}^*$ and $\boldsymbol{\Sigma}^a = \boldsymbol{M}^{-1}\boldsymbol{\Sigma}^*(\boldsymbol{M}^{-1})^T$. For example, $\mu_1^* = \mu_1^a$ and $\mu_k^* = \mu_k^a - \mu_1^a$ for $k > 1$.

Further, if $\boldsymbol{M}^c$ is the $(K-1)\times K$ matrix formed by deleting the first row of $\boldsymbol{M}$, then $\boldsymbol{\Sigma}^c = \boldsymbol{M}^c\boldsymbol{\Sigma}^a(\boldsymbol{M}^c)^T$.

C Choice of prior for heterogeneity variances

Evidence-based priors

The evidence-based priors for contrast heterogeneity variances [20] are of the form

$$\log\left(\sigma^{c2}\right) \sim N(m^c, s^{c2}) \tag{14}$$

and we call this the target prior distribution for σ^{c2}. Different evidence-based priors are used according to the type of outcome (objective, semi-objective or subjective) and treatment comparison (pharmacological vs. placebo/control, pharmacological vs. pharmacological, or non-pharmacological). The example considered in Section 6 has a semi-objective outcome and pharmacological vs. placebo/control comparisons, so our target prior distribution has $m^c = -3.02$, $s^c = 1.85$. We use the same target prior distribution for the hypothetical data.

Models 3 and 4 also involve arm heterogeneity variances σ^{a2}. We choose a target prior

$$\log\left(\sigma^{a2}\right) \sim N(m^a, s^{a2}). \tag{15}$$

In the absence of empirical evidence about this distribution, we choose the target mean $m^a = 0$ and the target standard deviation $s^a = 2$. The choice $m^a = 0$ expresses a view that the prior median of the arm heterogeneity variance σ^{a2} is 1, a value that we regard as quite large: considering the distribution of the odds of an event in the reference arm, it means for example that if the median is 0.25 (20% probability of an event) then 50% of studies lie between 0.08 and 0.80 (7% and 44% probability of an event).

Principles for selecting priors

We select the priors using the following principles.

1. All the CH models in this paper are directly specified in terms of σ^{c2}, so we use the target prior distribution directly.

2. In the NCH models, the priors for $\boldsymbol{\Sigma}^c$ and $\boldsymbol{\Sigma}^a$ should be symmetrical across treatments.

3. In the NCH models, we choose inverse Wishart priors for $\boldsymbol{\Sigma}^c$ and $\boldsymbol{\Sigma}^a$ that yield marginal priors for the contrast heterogeneity variances σ^{c2}

that are close to the target prior distribution. Specifically, we relate $\log \sigma^{c2}$ to $\boldsymbol{\Sigma}^c$ or $\boldsymbol{\Sigma}^a$ and match its mean exactly, and its variance as closely as possible, to the target prior distribution.

We use $W_d(\boldsymbol{R}, \nu)$ to denote the d-dimensional Wishart distribution with ν degrees of freedom and mean $\nu \boldsymbol{R}^{-1}$ [24]. We use several results about the Wishart distribution given in Supplementary Appendix D.

Symmetrical prior for models 2 and 3 NCH

For models 2 and 3 NCH, we require a prior for the $(K-1) \times (K-1)$ matrix $\boldsymbol{\Sigma}^c$. We specify

$$(\boldsymbol{\Sigma}^c)^{-1} \sim W_{K-1}(\boldsymbol{R}^c, \nu^c) \tag{16}$$

where we need to choose the integer $\nu^c \geq K-1$ and the $(K-1) \times (K-1)$ matrix $\boldsymbol{R}^c$.

To do this, we first work out the prior implied by (16) for the heterogeneity variance of the contrast of treatment k with treatment 1, Σ^c_{kk}. Let $\boldsymbol{e}_k$ be a row vector with a 1 in position k and zeroes elsewhere, and of dimension implied by the context. We can write $\Sigma^c_{kk} = \boldsymbol{e}_k \boldsymbol{\Sigma}^c \boldsymbol{e}_k^T$. Using result 6 in Supplementary Appendix D with $\boldsymbol{\Sigma} = \boldsymbol{\Sigma}^c$, $d = K-1$ and $\boldsymbol{N} = \boldsymbol{e}_k$ gives

$$(\Sigma^c_{kk})^{-1} \sim Ga\left(\frac{1}{2}(\nu^c - K + 2), \frac{1}{2} R^c_{kk}\right). \tag{17}$$

We next work out the prior implied by (16) for the heterogeneity variance of the contrast of treatment k' with treatment k, where $k' > k > 1$. This contrast heterogeneity variance is $\Sigma^c_{kk} - 2\Sigma^c_{kk'} + \Sigma^c_{k'k'}$ and may be written $(\boldsymbol{e}_k - \boldsymbol{e}_{k'})\boldsymbol{\Sigma}^c(\boldsymbol{e}_k - \boldsymbol{e}_{k'})^T$. Using result 6 in Supplementary Appendix D with $\boldsymbol{\Sigma} = \boldsymbol{\Sigma}^c$, $d = K-1$ and $\boldsymbol{N} = \boldsymbol{e}_k - \boldsymbol{e}_{k'}$ gives

$$(\Sigma^c_{kk} - 2\Sigma^c_{kk'} + \Sigma^c_{k'k'})^{-1} \sim Ga\left(\frac{1}{2}(\nu^c - K + 2), \frac{1}{2}(R^c_{kk} - 2R^c_{kk'} + R^c_{k'k'})\right). \tag{18}$$

The principle of symmetry between treatments requires the right hand sides of equations (17) and (18) to be equal for all k, k'. This holds for (17) if all R^c_{kk} equal a constant, f^c say, and for (18) if all $R^c_{kk'}$ equal $\frac{1}{2} f^c$. Hence $\boldsymbol{R}^c = f^c \boldsymbol{P}_{K-1}(\frac{1}{2})$ and

$$(\boldsymbol{\Sigma}^c)^{-1} \sim W_{K-1}(f^c \boldsymbol{P}_{K-1}(\frac{1}{2}), \nu^c). \tag{19}$$

Equation (19) is the generic symmetrical prior for models 2 and 3 NCH.

Target prior for models 2 and 3 NCH

We now match this symmetrical prior to the target prior.

Using result 7 in Supplementary Appendix D with $\boldsymbol{\Sigma} = \boldsymbol{\Sigma}^c$, $d = K - 1$ and $\boldsymbol{N} = \boldsymbol{e}_k$ gives

$$\mathrm{E}\left[\log \Sigma^c_{kk}\right] = \log\left(\frac{1}{2} f^c\right) - \psi\left(\frac{1}{2}(\nu^c - K + 2)\right) \tag{20}$$

where $\psi\,(.)$ is the digamma function. Matching this to the target prior mean m^c and solving gives

$$f^c = 2\exp\left\{m^c + \psi\left(\frac{1}{2}(\nu^c - K + 2)\right)\right\}. \tag{21}$$

We find that any choice $\nu^c > K - 1$ yields a prior less dispersed than the target prior, while choosing $\nu^c = K - 1$ gives a prior somewhat more dispersed than the target prior. We therefore take

$$\nu^c = K - 1 \tag{22}$$

as a somewhat conservative choice.

Symmetrical prior for model 4 NCH

For model 4 NCH, we require a prior for the $K \times K$ matrix $\boldsymbol{\Sigma}^a$. We specify

$$(\boldsymbol{\Sigma}^a)^{-1} \sim W_K(\boldsymbol{R}^a, \nu^a) \tag{23}$$

where we need to choose the integer $\nu^a \geq K$ and the $K \times K$ matrix $\boldsymbol{R}^a$. To make the prior symmetrical across treatments, we require Σ_{kk} to have the same prior for all k, and $\Sigma_{kk'}$ to have the same prior for all $k' \neq k$, with no constraint relating Σ_{kk} and $\Sigma_{kk'}$. It follows that $\boldsymbol{R}^a$ must be compound symmetrical, $\boldsymbol{R}^a = f^a \boldsymbol{P}_K(r^a)$ for some scalars f^a and r^a, and hence

$$(\boldsymbol{\Sigma}^a)^{-1} \sim W_K(f^a \boldsymbol{P}_K(r^a), \nu^a). \tag{24}$$

Equation (24) is the generic symmetrical prior for model 4 NCH.

We now show that the choices of priors for models 2-4 NCH are consistent, in that a prior for $\boldsymbol{\Sigma}^a$ of the form (24) in model 4 NCH implies a prior for $\boldsymbol{\Sigma}^c$ of the form (19) in models 2 and 3 NCH. We use result 3 from Supplementary Appendix D with $\boldsymbol{\Sigma} = \boldsymbol{\Sigma}^a$, $d = K$ and $\boldsymbol{N} = \boldsymbol{M}_c$ defined in Supplementary Appendix B. This shows that $(\boldsymbol{\Sigma}^{\boldsymbol{c}})^{-1} = (\boldsymbol{M}_c \boldsymbol{\Sigma}^{\boldsymbol{a}} \boldsymbol{M}_c^T)^{-1}$ has prior $W_{K-1}(f^a \boldsymbol{M}_c \boldsymbol{P}_K(r^a) \boldsymbol{M}_c^T, \nu^a - 1)$. It is easy to show $\boldsymbol{M}_c \boldsymbol{P}_K(r^a) \boldsymbol{M}_c^T = 2(1 - r^a)\boldsymbol{P}_{K-1}(\frac{1}{2})$. Thus the prior for $(\boldsymbol{\Sigma}^{\boldsymbol{c}})^{-1}$ is of the form (19) with

$$\nu^c = \nu^a - 1 \tag{25}$$

$$f^c = 2f^a(1 - r^a). \tag{26}$$

Target prior for model 4 NCH

Using result 6 from Supplementary Appendix D with $\boldsymbol{\Sigma} = \boldsymbol{\Sigma}^a$, $d = K$ and $\boldsymbol{N} = \boldsymbol{e}_k$ gives

$$(\Sigma_{kk}^a)^{-1} \sim Ga\left(\frac{1}{2}(\nu^a - K + 1), \frac{1}{2}f^a\right)$$

and hence

$$\mathrm{E}\left[\log \Sigma_{kk}^a\right] = \log\left(\frac{1}{2}f^a\right) - \psi\left(\frac{1}{2}(\nu^a - K + 1)\right). \tag{27}$$

We choose f^a by matching the above expression to the target mean m^a:

$$f^a = 2\exp\left(m^a + \psi\left(\frac{1}{2}(\nu^a - K + 1)\right)\right). \tag{28}$$

Next, comparing (27) with (20) and using (25) and (26),

$$\mathrm{E}\left[\log\left(\Sigma_{kk}^a\right)\right] - \mathrm{E}\left[\log\left(\Sigma_{kk}^c\right)\right] = -\log\left(2(1 - r^a)\right) \tag{29}$$

so that we can find r^a as

$$r^a \;\; = \;\; 1 - \frac{1}{2}\exp\left(m^c - m^a\right). \tag{30}$$

Finally, as for models 2 and 3, we find that any choice $\nu^a > K$ yields a prior for the contrast heterogeneity variance that is less dispersed than the target prior, while choosing $\nu^a = K$ gives a prior somewhat more dispersed than the target prior. We therefore take

$$\nu^a = K \tag{31}$$

as a somewhat conservative choice.

We can also show that $\mathrm{var}\left(\log\left(\Sigma_{kk}^a\right)\right) = \mathrm{var}\left(\log\left(\Sigma_{kk}^c\right)\right)$. Thus the Wishart distribution does not allow us to specify the spread of Σ_{kk}^a in model 3 NCH separately from the spread of Σ_{kk}^c.

Hong *et al.*, like us, took all inverse covariance matrices to be Wishart with $\nu^c = K - 1$ and $\nu^a = K$, but they took $R^c = 5(K-1)\boldsymbol{I}_{K-1}$ and $R^a = 5K\boldsymbol{I}_K$, giving distributions with means $5\boldsymbol{I}_{K-1}$ and $5\boldsymbol{I}_K$ respectively [3]. These distributions could be scaled to suit the problem in hand. However, they have the disadvantages: (1) for models 2 and 3, it gives different priors for different contrasts, so that results depend on the choice of reference treatment; (2) the priors for the contrast heterogeneity variance differ between models 2-4, meaning that comparisons between models may reflect differences in priors.

Priors for arm heterogeneity

For model 3 (both CH and NCH), we specify the prior for σ^a directly using the target prior (15).

For model 4 CH, we require a prior for ρ^a. Using equation (13) and taking logs gives $\log(\sigma^{a2}) = \log(\sigma^{c2}) - \log(2(1-\rho^a))$. To achieve target priors (14) and (15), we choose a prior for ρ^a independent from σ^{c2} such that $-\log(2(1-\rho^a))$ has mean $m^a - m^c$ and variance $\sigma^{a2} - \sigma^{c2}$. We choose to use beta prior $\rho^a \sim Be(a, b)$ and find by trial and error that $\rho^a \sim Be(60, 2)$ (a beta distribution with mean near 1) is suitable.

D Notes on the inverse Wishart prior

The following results are used in Supplementary Appendix C.

$\boldsymbol{W} \sim W_d(\boldsymbol{R}, \nu)$ means $\boldsymbol{W}$ is distributed like the $d \times d$ sum-of-squares matrix for a sample of ν observations from a d-dimensional MVN distribution with mean $\mathbf{0}$ and variance $\boldsymbol{R}^{-1}$. Thus $p(\boldsymbol{W}) \propto |\boldsymbol{R}|^{\nu/2}|\boldsymbol{W}|^{(\nu-d-1)/2} \exp\left(-\frac{1}{2}tr(\boldsymbol{RW})\right)$ with $\nu > d-1$. Further, $\boldsymbol{\Sigma} = \boldsymbol{W}^{-1}$ is inverse Wishart, written $\boldsymbol{\Sigma} \sim W_d^{-1}(\boldsymbol{R}^{-1}, \nu)$. The Wishart matrix $\boldsymbol{W}$ and inverse Wishart matrix $\boldsymbol{\Sigma}$ have the following properties [39]:

1. $\mathrm{E}[\boldsymbol{W}] = \nu\boldsymbol{R}^{-1}$.

2. $\mathrm{E}[\boldsymbol{\Sigma}] = (\nu - d - 1)^{-1}\boldsymbol{R}$ provided $\nu > d + 1$.

3. Transformation. If $d^* \leq d$ and $\boldsymbol{N}$ is a $d^* \times d$ matrix of rank d^*, then $\boldsymbol{NWN}^T \sim W_{d^*}((\boldsymbol{NR^{-1}N}^T)^{-1}, \nu)$ and $(\boldsymbol{N\Sigma N}^T)^{-1} \sim W_{d^*}(\boldsymbol{NRN}^T, \nu - d + d^*)$.

4. Let $G \sim Ga(\alpha, \beta)$ refer to the gamma distribution with mean α/β and variance α/β^2. Then $\mathrm{E}[\log G] = \psi(\alpha) - \log(\beta)$ where (as in Appendix C) $\psi(.)$ is the digamma function.

5. $W_1(R, \nu)$ is the same distribution as the scaled chi-squared distribution $\frac{1}{R}\chi^2_\nu$ which in turn is the same as the gamma distribution $Ga(\frac{1}{2}\nu, \frac{1}{2}R)$.

6. Transformation of inverse Wishart to a scalar: with the special case of $d^* = 1$ and $\boldsymbol{N}$ a row vector, we get a scalar random variable $(\boldsymbol{N}\Sigma\boldsymbol{N}^T)^{-1} \sim Ga\left(\frac{1}{2}(\nu - d + 1), \frac{1}{2}\boldsymbol{NRN}^T\right)$. This is also given by [40].

7. Hence $\mathrm{E}\left[\log\left(\boldsymbol{N\Sigma N}^T\right)\right] = \log\left(\frac{1}{2}(\boldsymbol{NRN}^T)\right) - \psi\left(\frac{1}{2}(\nu - d + 1)\right)$.

E Stata code for data analysis

```
// Analyses using -network- version 1.6.0
//     which is available from
//     http://www.homepages.ucl.ac.uk/~rmjwiww/stata/
// Evidence-based prior for sigma^c2
//     has mean -3.02 and SD 1.85
//     hence precision 1/1.85^2=0.2922

use data5b, clear // or other data
network setup d n, studyvar(study)

* Model 1 CH
network bayes, name(Model1_CH) model(1) commonhet    ///
    sigC2prior(dlnorm(-3.02,0.2922))                 ///
    quit burnin(5000) updates(200000) thin(20)

* Model 2 CH
network bayes, name(Model2_CH) model(2) commonhet    ///
    sigC2prior(dlnorm(-3.02,0.2922))                 ///
    quit burnin(5000) updates(200000) thin(20)

* Model 2 NCH
network bayes, name(Model2_NCH) model(2) nocommonhet ///
    logsigC2mean(-3.02)                              ///
    quit burnin(5000) updates(200000) thin(20)

* Model 3 CH
network bayes, name(Model3_CH) model(3) commonhet    ///
    sigC2prior(dlnorm(-3.02,0.2922))                 ///
    sigAprior(dlnorm(0,1))                           ///
    quit burnin(5000) updates(200000) thin(20)

* Model 3 NCH
network bayes, name(Model3_NCH) model(3) nocommonhet ///
    logsigC2mean(-3.02)                              ///
    sigAprior(dlnorm(0,1))                           ///
    quit burnin(5000) updates(200000) thin(20)

* Model 4 CH
network bayes, name(Model4_CH) model(4) commonhet    ///
```

```
    sigC2prior(dlnorm(-3.02,0.2922))                  ///
    rhoprior(dbeta(25,1))                              ///
    quit burnin(5000) updates(200000) thin(20)

* Model 4 NCH
network bayes, name(Model4_NCH) model(4) nocommonhet ///
    logsigC2mean(-3.02)                                ///
    logsigAmean(0)                                     ///
    quit burnin(5000) updates(200000) thin(20)
```

F Definitions of A-MAR and C-MAR

Recall that $y^c_{ikk'}$ is the estimated contrast between arms k and k' in study i. Then C-MAR states that

$$p(R_i = r_i | \{y^c_{ikk'} : 1 \leq k < k' \leq K\}) = p(R_i = r_i | \{y^c_{ikk'} : k, k' \in r_i, k < k'\})$$

and A-MAR states that

$$p(R_i = r_i | \{y^a_{ik} : 1 \leq k \leq K\}) = p(R_i = r_i | \{y^a_{ik} : k \in r_i\}).$$

Analogous to these definitions of A-MAR and C-MAR, we define "C-MCAR":

$$p(R_i = r_i | \{y^c_{ikk'} : 1 \leq k \leq k' \leq K\}) = p(R_i = r_i).$$

and "A-MCAR":

$$p(R_i = r_i | \{y^a_{ik} : 1 \leq k \leq K\}) = p(R_i = r_i).$$

G Does MAR imply MCAR in a CB likelihood?

We first consider the case when all studies are two-arm studies. In this case, we show that the C-MAR assumption does not imply the C-MCAR assumption. C-MAR implies $p(R_i = \{k, l\}|Y) = p(R_i = \{k, l\}|y^c_{ikl}) = p_{ikl}(y^c_{ikl})$ say. These probabilities must sum to one for each i: $\sum_{k,l} p_{ikl}(y^c_{ikl}) = 1$. For a network of XY, XZ and YZ trials, we could have

$$\begin{aligned} p_{iXY}(y^c_{iXY}) &= 1/3 + \gamma y^c_{iXY} \\ p_{iYZ}(y^c_{iYZ}) &= 1/3 + \gamma y^c_{iYZ} \\ p_{iXZ}(y^c_{iXZ}) &= 1/3 - \gamma y^c_{iXZ} \end{aligned}$$

in which the sum does not depend on the data since $y^c_{iXZ} = y^c_{iXY} + y^c_{iYZ}$. This is therefore a valid C-MAR mechanism that (if $\gamma \neq 0$) is not C-MCAR.

In the special case of a star network (that is, one in which all treatments are compared only with the reference treatment), however, the sum runs over $k = 1$ and $l = 2, \ldots, K$. Each observation from an arm $l > 1$ occurs only once in the sum, so cancellation can not occur: therefore the probabilities cannot depend on the data and must be constants. Thus in a star network, any C-MAR mechanism is C-MCAR.

The above example was rather artificial. A simpler example of a mechanism that is C-MAR but not C-MCAR is possible using multi-arm studies. Suppose all studies include arms X and Y but only some studies include arm Z. In this case, C-MAR holds if the design depends only on the X-Y contrast, and this is not a C-MCAR mechanism.

H Extra references in supplementary materials

[39] Rao CR. *Linear Statistical Inference and its Applications*. Wiley Series in Probability and Statistics. John Wiley & Sons, Inc.; Hoboken, NJ, USA; 1973.

[40] Alvarez I, Niemi J, Simpson M. Bayesian inference for a covariance matrix. arXiv:1408.4050v2 [stat.ME]. https://arxiv.org/abs/1408.4050v2.

Figure S1: Hypothetical data sets: estimated treatment contrasts Y and Z vs. X, showing posterior medians of the log odds ratio with 95% credible intervals; vertical lines show the true values seen in each individual data set. CH=common heterogeneity (solid lines); NCH=non-common heterogeneity (dashed lines).

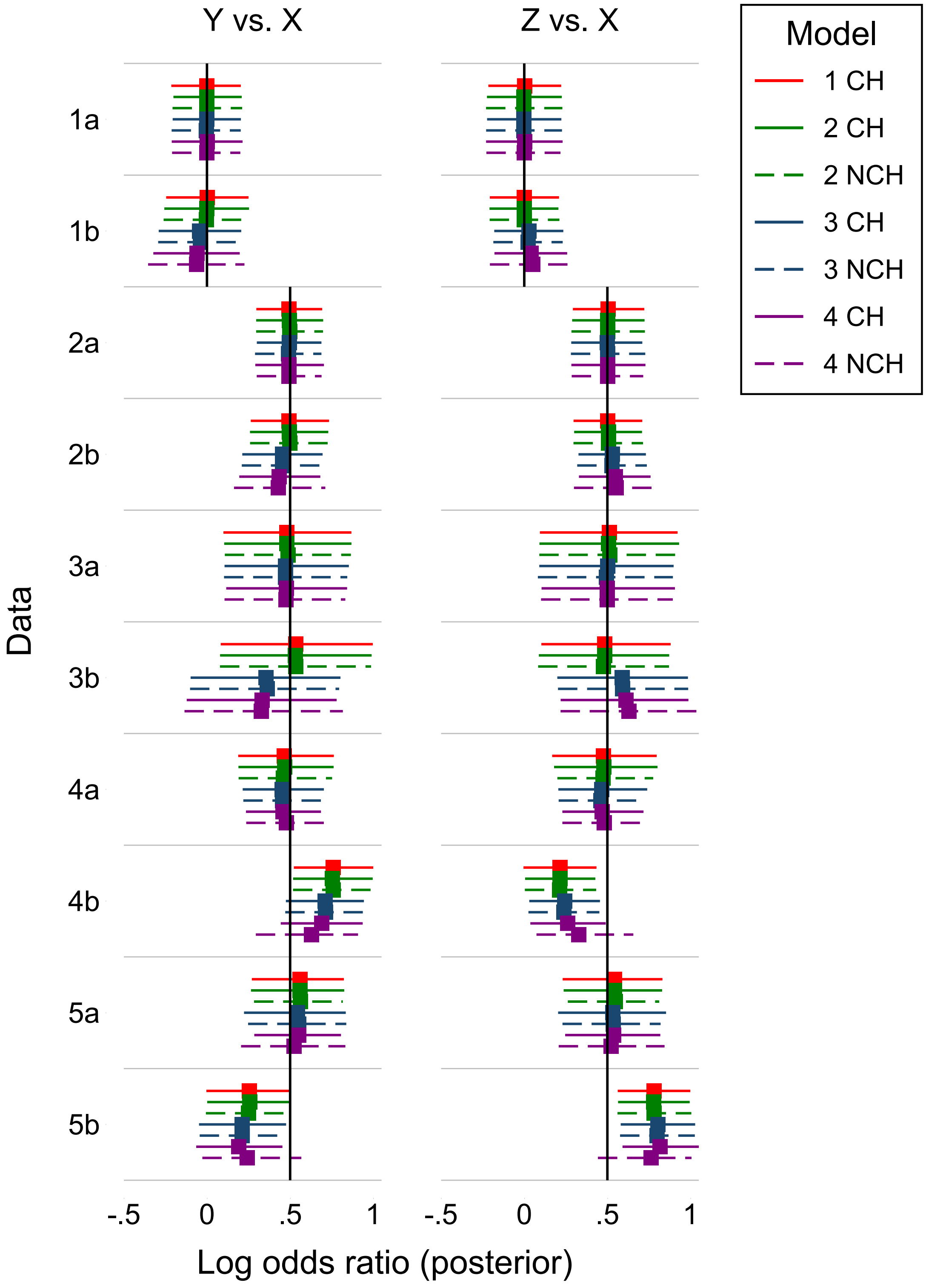

Figure S2: Hypothetical data sets: estimated log contrast heterogeneity standard deviations (SDs), showing posterior medians with 95% credible intervals. CH=common heterogeneity (solid lines); NCH=non-common heterogeneity (dashed lines). Estimates for contrast Z vs. X and Z vs. Y are only shown for NCH models; for CH models they are the same as for contrast Y vs. X.

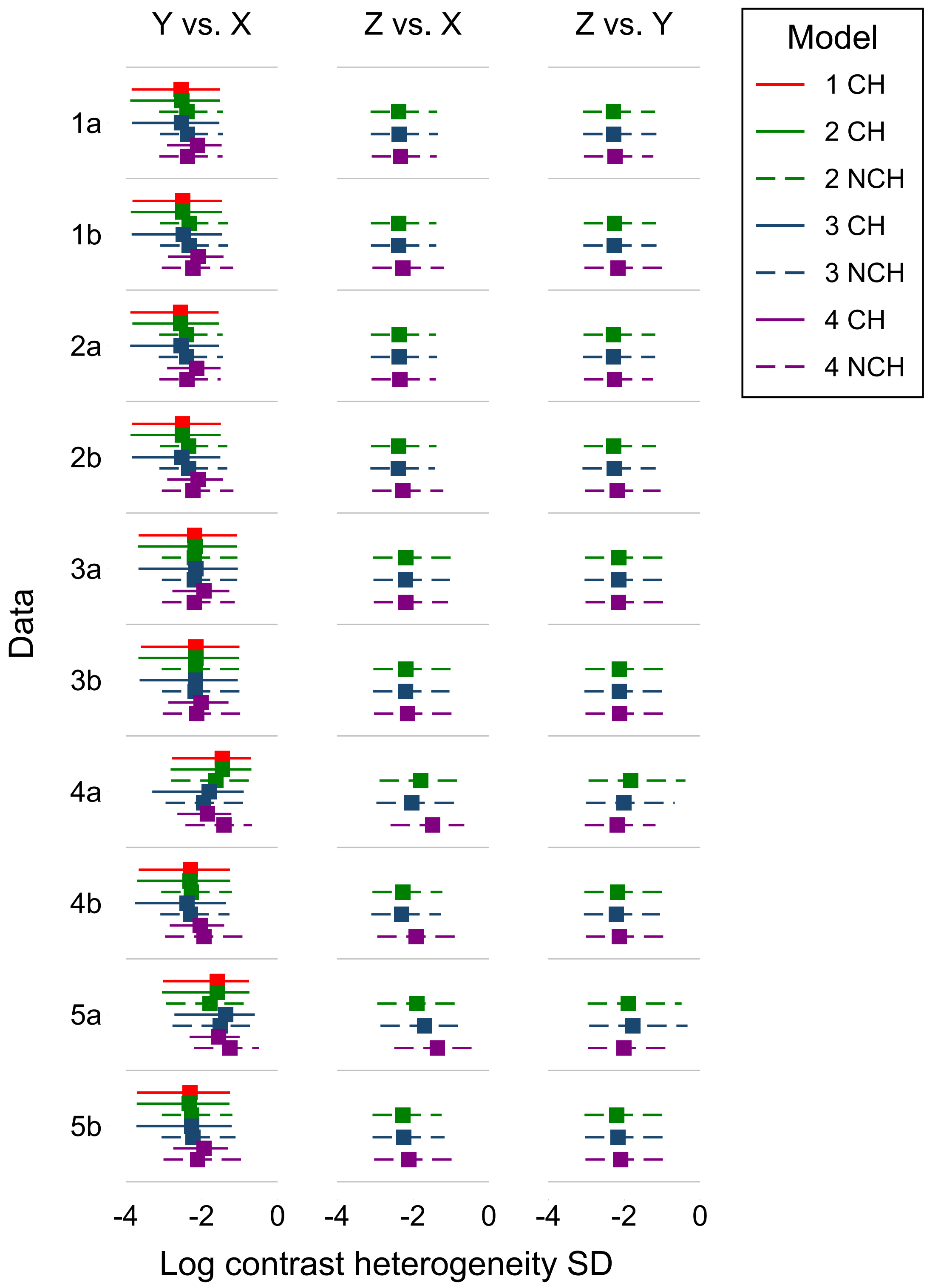

Figure S3: Hypothetical data sets: estimated log arm heterogeneity standard deviations (SDs), showing posterior medians with 95% credible intervals. CH=common heterogeneity (solid lines); NCH=non-common heterogeneity (dashed lines). Estimates for arm Y and Z are only shown for model 4 NCH; for model 4 CH models they are the same as for arm X.

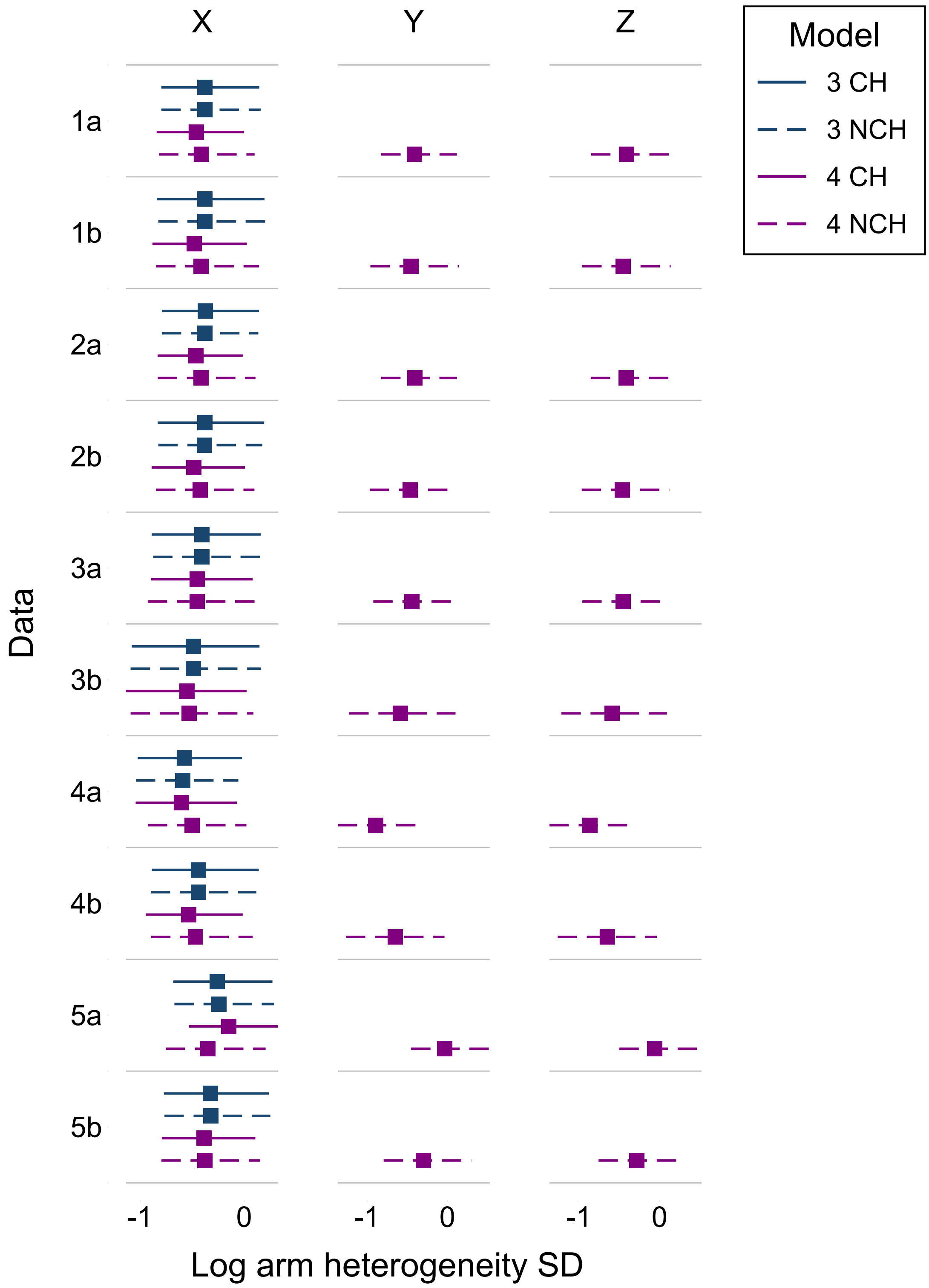

Figure S4: Hypothetical data sets: priors for all parameters, showing medians with 95% credible intervals. CH=common heterogeneity (solid lines); NCH=non-common heterogeneity (dashed lines).

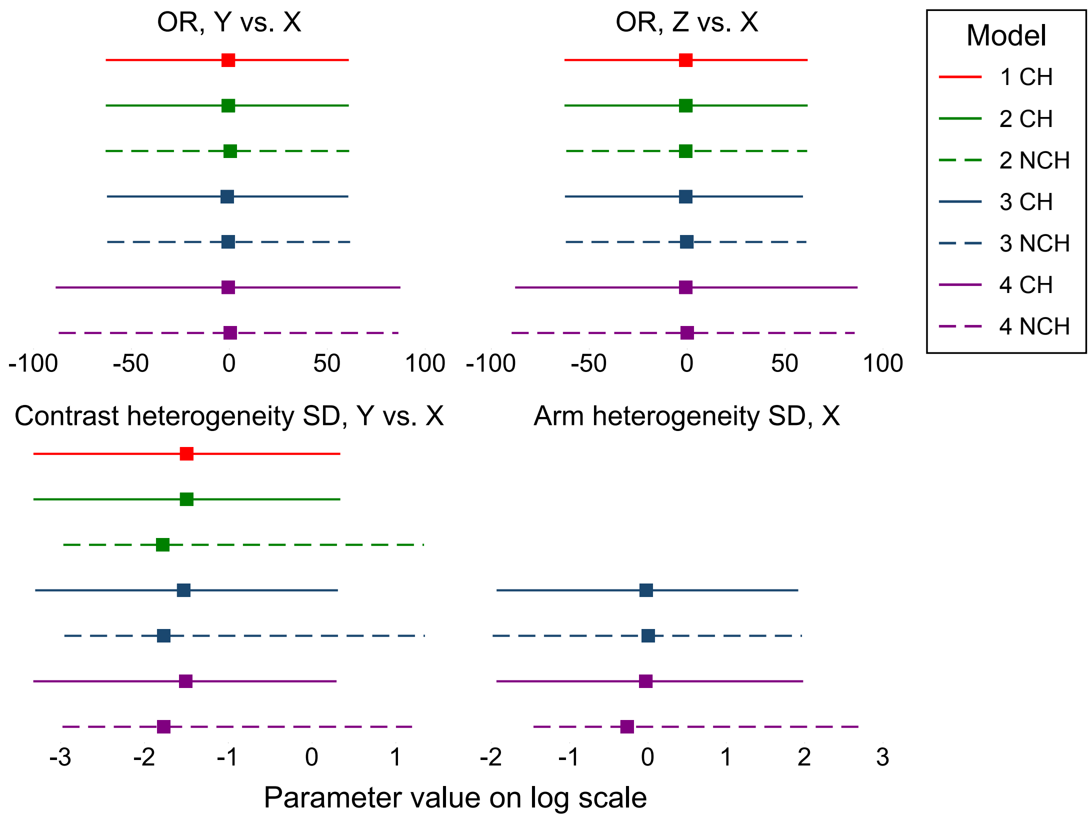

Figure S5: Inhaled corticosteroids network: structure

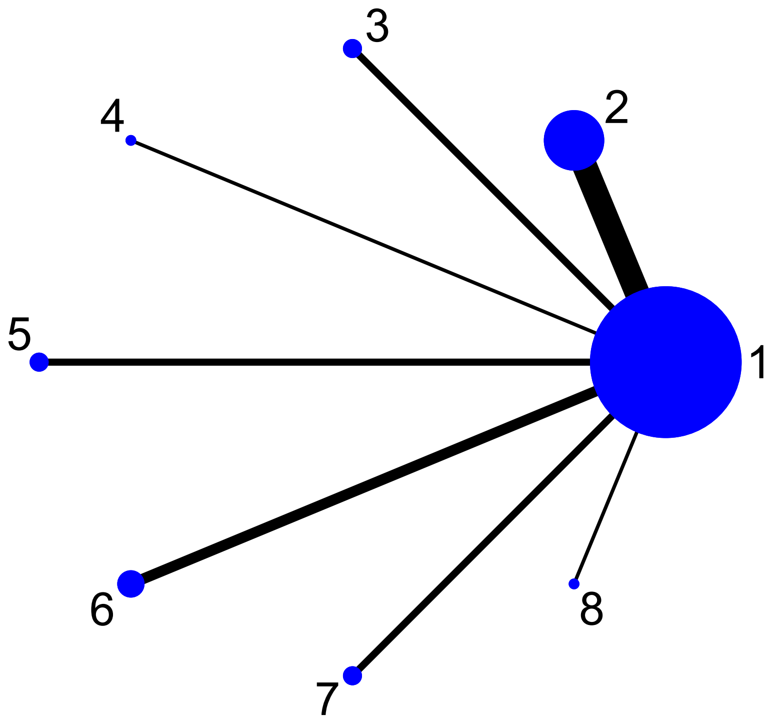

Supplement: Supplementary file 1 — SIM_8360‐Supp‐0001‐ABCB_supplementary.pdf [file SIM-38-5197-s001.pdf]
